# Using Vector Autoregression Modeling to Reveal Bidirectional Relationships in Gender/Sex-Related Interactions in Mother–Infant Dyads

**DOI:** 10.3389/fpsyg.2020.01507

**Published:** 2020-08-05

**Authors:** Elizabeth G. Eason, Nicole S. Carver, Damian G. Kelty-Stephen, Anne Fausto-Sterling

**Affiliations:** ^1^Department of Mathematics and Statistics, Grinnell College, Grinnell, IA, United States; ^2^Department of Psychology, University of Cincinnati, Cincinnati, OH, United States; ^3^Department of Psychology, Grinnell College, Grinnell, IA, United States; ^4^Department of Molecular Biology, Cell Biology and Biochemistry, Brown University, Providence, RI, United States

**Keywords:** development, dyads, mother–infant, gender/sex, vector autoregression

## Abstract

Vector autoregression (VAR) modeling allows probing bidirectional relationships in gender/sex development and may support hypothesis testing following multi-modal data collection. We show VAR in three lights: supporting a hypothesis, rejecting a hypothesis, and opening up new questions. To illustrate these capacities of VAR, we reanalyzed longitudinal data that recorded dyadic mother–infant interactions for 15 boys and 15 girls aged 3 to 11 months of age. We examined monthly counts of 15 infant behaviors and 13 maternal behaviors (Seifer et al., 1994). VAR models demonstrated that infant crawling predicted a subsequently close feedback loop from mothers of boys but a subsequently open-ended, branched response from mothers of girls. A different finding showed that boys’ standing independently predicted significant later increases of four maternal behaviors: rocking/jiggling, lifting, affectionate touching, and stimulation of infant gross-motor activity. In contrast, crawling by girls led mothers to later decrease the same maternal behaviors. Thus, VAR might allow us to identify how mothers respond differently during daily interactions depending on infant gender/sex. The present work intends to mainly showcase the VAR method in the specific context of the empirical study of gender/sex development.

## Introduction

Bidirectional relationships are classically the stock in trade of modern psychological theorizing (e.g., Gottlieb, 2007a, b). For instance, developmental psychology regularly appeals to the concept of the “active child.” This infant is not simply the passive recipient of environmental influences but is also an assertive agent bringing their own interests and impulses to the interactive table. As infants develop language and symbolic expression, they begin to translate the sensory information stored in their nervous systems during infancy (so-called presymbolic representation; Beebe and Lachmann, 1994) into behaviors and preferences that in turn invite specific changes in caregivers’ behaviors. We accept as uncontroversial the idea that development is a perpetual give and take between developing organism and context.

However, despite insisting on the crucial role of bidirectional relationships underlying the development of complex systems (Ashby, 1956), as a field, psychology has been slow to incorporate the formal analyses that would give bidirectional hypotheses appropriate mathematical framing. The prevailing analytical strategies are *t*-tests, analysis of variance (ANOVA), and linear regression. These are profoundly robust strategies, but for the most part, they require psychologists to choose one variable as a predictor or grouping variable and the other variable as the dependent measure. In correlational research, psychologists regularly encounter the challenge of not knowing which variable is causing the other (e.g., “the directionality problem”; Gazzaniga, 2018). So, psychologists may sometimes make do with modeling development in piecemeal fashion, treating this or that variable by turn as the grouping variable or the dependent variable—this point applies equally well to even very elegant approaches such as hierarchical linear regression. In this paper, we are highlighting that these traditional statistical approaches do not actually make bidirectional relationships explicit and testable as part of an inferential statistical model.

Nevertheless, statistical models for modeling bidirectional effects—and so for giving full statistical leverage to these elegant ideas—have existed for decades. We have in mind for the present manuscript a method called “vector autoregression” (VAR; Sims, 1980) that, despite having enjoyed rich elaboration and wide application over the past few decades (Lutkepohl, 2005), has remained out of the view of some developmental psychologists. This incomplete appearance of VAR to psychologists is likely due to the fact that these methods arose in econometrics and have only appeared in a few subfields of psychology also interested in bidirectional relationships (Roebroeck et al., 2005; Billinger et al., 2014; van Winkel et al., 2017; Xu et al., 2020). In this paper, we will showcase VAR for a developmental–psychological audience.

For the developmental psychologist interested in bidirectional relationships between, say, infant and caregiver, a central question is: What are the possible ways to understand the complex dyadic choreography? To illustrate this general formal challenge, we take the specific example of gender/sexed behaviors in infants. Gender/sex differentiation results from a complex interplay, beginning prenatally and extending well past adolescence, between nature and nurture. Gender/sexed toy, color, and play preferences take shape during infancy (O’Brien et al., 1983; O’Brien and Huston, 1985; Campbell et al., 2000; Serbin et al., 2001), well before toddlers (roughly 2.5–3.5 years) begin to exhibit gender/sexed knowledge (Bussey and Bandura, 1999; Martin et al., 2002, 2004; Bandura and Bussey, 2004). Furthermore, caregivers differentially employ touch-based interactions according to infants’ genital sex (Fausto-Sterling et al., 2015), and infants use sound and sight cues to distinguish adult males from adult females (Ruble et al., 2006; Fausto-Sterling et al., 2012a, b) while developing the ability to recognize culturally specific gender/sexed adult behaviors (Eichstedt et al., 2002). Studying individual relationships singly omits not just the multifactorial aspect of development but also the bidirectional relationships between the actively developing child and the responsive environment. The complexity and bidirectionality at play in gender/sex differentiation make it immensely challenging to study gender/sex development solely using narrowly constrained traditional hypothesis testing.

Importantly and perhaps surprisingly, VAR has already been hiding in plain sight at the heart of recent methods applied by developmental psychology, such as state-space modeling (SSM), lag-sequential analysis, and dynamic structural equation modeling (DSEM). The first two analyses draw immediately on data from continuous coding programs such as Noldus Observer. For example, Sung et al. (2013) examined vocal exchanges between mothers and sons, compared to mothers and daughters by calculating how frequently—within a 1-s lag—a vocal event from the mother is followed by an infant vocal event or vice versa. Crucially, both SSM and DSEM are methods that use repeated measurements to explore how lag-sequential relationships among different measurements unfold across a theoretically driven network of latent variables; SSM and DSEM do effectively similar work in different form. At root SSMs and DSEMs are each rewritable in terms of the other: they are both VAR models enhanced with latent variables (Hamaker et al., 2018; Hunter, 2018). Latent variables are computational tokens that represent the underlying constructs as related but separate from the measurements that operationalize these constructs. SSMs have focused less on a singular cause of an individual behavior and more on the dynamics of dyadic behaviors, both during short-term interactions and as they evolve over longer periods of time (Hollenstein, 2007, 2013). SSMs can reveal the short-term structure of a dyadic system and, through re-analysis over longer time periods, can reveal dynamic changes in system structure (Hollenstein and Tsui, 2019).

In a sense, what we aim to do here is to showcase the important option to take the relatively agnostic step to remove the latent variables and explore the VAR relationships free of theoretical preconceptions. At least in early explorations, research into gender/sex identity and/or gender/sex expression could benefit from such an agnostic approach to probing relationships among variables over time—that is, how each variable’s current values influence later values of itself and other variables. The non-developmental psychological literature already recognizes VAR as a data-driven approach to estimating bidirectional relationships across time, and there are certainly ways to constrain this data-driven approach with firmer theoretical frameworks (Chen et al., 2011). Even though we take the well-demonstrated lesson that exploratory data analysis can be a poor way to go about rejecting null hypotheses (Simmons et al., 2011), a field such as gender/sex development has at least two major reasons to take a more exploratory view: first, cultural belief systems have biased theorizing on the very biological roots of sex, let alone gender/sex (Richardson, 2013). Second, the results have encouraged debate as to whether even our most cutting-edge theories of development manage so far to predict or explain anything but the most traditional gender/sex outcomes and the simplest unidirectional effects (Liben and Coyle, 2017; Richardson, 2017; Bentley et al., 2019; Hyde et al., 2019). In short, theory-driven hypothesis is generally the best practice, but when the current theorizing bends under cultural belief systems toward only predicting the most traditional or most simplified outcomes, theories may remain relatively mute on what more complex, bidirectional relationships we should predict at the outset. So, we believe that there remains a place for exploratory views for what bidirectional relationships our current theories may not be ready to predict. Indeed, we follow Hyde et al. (2019) in using the term “gender/sex” rather than either “gender” or “sex” alone to respect the inseparability of biological and the cultural—and so the inadequacy of scientific approaches that leave them separate.

For gender/sex development or indeed for any field of psychological research waiting for theoretical developments to catch up to the diversity in evidence, VAR offers a valuable tool for both exploring and testing bidirectional gender/sex-based effects between mother–infant dyads. We use VAR modeling to examine scaffolding of certain motor behaviors during the first year of development in infants of different gender/sexes. Despite agreement that boys and girls reach motor milestones on the same schedule, reports abound of differences in activity level, and of motor skills such as handling or throwing objects, and athletic achievements such as running and jumping that emerge in mid to late childhood (e.g., Boatella-Costa et al., 2007). For many of these skills, gender/sex differences emerge on the time scale of months to years. We will focus only on the time scale of months and examine how mother and infant press one another into patterns of behavior across time. VAR may allow us to identify (and subsequently test the importance of) possible stepping stones built at the start of life over months of maternal/infant interactions through subsequent iterations of particular motor skills.

Vector autoregression yields a wider view for modeling bidirectional relationships, quite unlike standard analysis of variance (ANOVA) and unlike the more elegant models resting upon latent-variable networks. Whereas an ANOVA will only test whether changes in one set of variables produce changes in one variable at one time scale, VAR offers the possibility of testing whether each variable might be associated with later changes of all other variables at many time scales. Removing the latent variables as in SSM and DSEM moors our statistical view more firmly in the measurements. While that removal does risk overemphasis of measurement error (Hunter, 2018), the latent variables reflect just that imprint of theory that risks overemphasizing our own biases and blind spots. To the degree that developmental–psychological outcomes are more complex than one variable influencing another at one time, VAR offers a wider view of the more complex, bidirectional relationships and at more time scales—particularly when VAR is unfettered from the latent variables of SSMs and DSEMs. However, to the degree that wider view rests on a wider set of statistical relationships, VAR poses a somewhat daunting abundance of structure—daunting because it amounts to a new thicket of statistical detail.

To focus our attention amidst this abundance of structure, we present this method in three different lights. To aid reader understanding, we sketch three types of findings that illustrate some of the ways that VAR might inform developmental research: (1) expanding the scope of known effects, (2) uncovering significant evidence against literature-motivated hypotheses, and (3) uncovering so-far non-obvious effects that our theoretical perspectives have not explicitly predicted (e.g., Gottlieb, 2007a, b). None of these outcomes are unique to VAR, but we list them to help organize our example and show how VAR can generate all three of these outcomes in a framework that models bidirectional effects within a dyad. Before we proceed any further, it is important for us to insist that the present work aims to showcase the method and not to make an argument. To provide a better sense of what the model can do, we include empirical data as an example of what the modeling strategy might uncover. A methods paper without an empirical example risks asking too much of the reader’s imagination. We present the empirical examples to showcase the type of results that VAR produces.

Here are some specific examples of each type of hypothesis that our subsequent VAR will test in this paper: (1) Maternal pointing gestures are important in vocabulary acquisition (Pan et al., 2005). Indeed, a plausible reading of the literature suggests that during the first 8–10 months, maternal pointing aids the development of infant pointing (Kishimoto, 2017). Thereafter, infant pointing, an action one set of authors refer to as an epistemic request (Kovács et al., 2014), leads to maternal speech that teaches vocabulary. But does maternal pointing only have an effect in the realm of language acquisition? Using VAR, we test the possibility that it may also be important during motor development. (2) VAR will be used to test the claim that maternal behaviors providing gross-motor stimulation might encourage more physical activity from boys than from girls (e.g., Smith and Lloyd, 1978; Landerholm and Scriven, 1981). Earlier work showing that during infancy mothers engage in more stimulatory touch with sons compared to daughters also raises the possibility that this differential leads to boys’ higher physical activity levels (Fausto-Sterling et al., 2015). (3) VAR will be used to discover or uncover maternal responses to infant behaviors that differ by gender/sex of the infant above and beyond any infant response to maternal input. Hence, we aim to showcase VAR as a method offering, first, support from an available data set for known effects, second, rejection of perhaps intuitive hypotheses, and, finally, opening up altogether new and unexpected developmental relationships.

## Materials and Methods

### Data Source and Collection

Full details concerning the authors’ observation and coding methods may be found in published work (Sung et al., 2013; Fausto-Sterling et al., 2015).

### Participants

Thirty families were matched for infant age and completeness of biweekly time samples from 50 families originally observed and taped by Seifer et al. (1994) for a study of infant temperament. The mothers were all first-time mothers, white, predominately middle and working class, married women from Rhode Island, aged, on average, 29.1 years (SD = 4.2, range 22.2–36.9) on their child’s birthdate. Four-factor SES averaged 1.96 (SD = 0.75, range 1–4). All children had been full-term babies. The original, in-home videotapes were collected with permission using procedures sanctioned at the time by an Institutional Review Board. Reuse of the videos for the current study was permitted under the terms of the original consent forms, but underwent a second, specific review and approval by the Brown University Institutional Review Board.

### Procedure

Mother–infant interactions in the infants’ homes were videotaped with minimal intrusion weekly for about an hour with minimal intrusion for 40 weeks. During each hour-long session, mother–infant dyads spent at least 10 min playing together (Seifer et al., 1994). For most observations, the 10-min play period occurred in a contiguous block of time, but in some cases, extended play might be broken. For example, a mother might play with her infant about 5 min, then change a diaper and then return to playing with her infant. The mother–infant play scenes were randomly distributed throughout early, middle, and end parts of the hour-long session. Present analyses used randomly selected 5-min segments from two play sessions per month for months 3–12, giving 20 observations per infant. This selective time window allowed fine-grained behavioral coding and followed current evidence that thin slices of time are appropriate for behavioral coding of social interactions (Murphy, 2005; Adolph and Robinson, 2011). Although it might seem that more repeated measures would be better, VAR modeling is actually best served by short-term data sets for two reasons: (1) VAR only models short-range relationships (i.e., relationship across small numbers of lags) and so requires residuals without long-range temporal correlations and (2) long-term raw measures of human behavior are known to embody precisely the long-range temporal correlations that would make VAR models fail to converge properly (Van Orden et al., 2003; Ihlen and Vereijken, 2010). Furthermore, we mitigated any effect of individual weeks yielding unrepresentative data by pooling the weekly data by month. This pooling had the additional benefit of better ensuring that the data submitted to VAR modeling would have equal spacing.

### Behavioral Coding

We performed second-by-second behavioral coding used Observer XT 7 software (Noldus Information Technology, Wageningen, Netherlands) using codes in Table 1. The Noldus program allows batch exporting of data to SPSS. In this study, we focused on two renderings into SPSS—for each code—mean event frequency and mean duration of event. From SPSS, all codes used in this study were imported into R and aggregated by month.

**TABLE 1:** Code definitions.

Infant Behaviors	Definition
Stand (Object support)	Infant stands while holding onto something for support
Stand (Mother help)	Infant stands and the mother assists by holding the infant’s hands or trunk
Stand Independently	Infant stands without holding onto mother or any other object
Sit (Object support)	Infant sits, typically in a seat with back support
Sit (Mother help)	Infant sits with support from mother
Sit Independently	Infants sits on their hips or bottom
Lie (All)	Includes lying still, rolling over, and lying kicking
Lie Still	Infant lies down and does not move
Babble	All non-distressed, syllabic vocalizations where coder cannot distinguish clear words
Cry	Infant’s vocalizations indicate distress during vocalization
Reach	Infant extends arm to grasp/get an object or to offer or to show an object
Crawl	Infant moves by some means other than walking
**Play Frame (dyadic play)**	
Play (Passive)	Infant plays with objects (toys, other play objects) without mother’s direct assistance
Play (Motor-Social)	The mother plays using gestures alone (i.e., no toys)
Play (Object)	The mother plays with an object jointly with the infant
**Maternal Behaviors**	
Rocks or jiggles infant	The mother rocks, jiggles, or moves the infant rhythmically
Lifts infant	Mother lifts the infant into the air as a kind of motor play type
Assists locomotion	Mother holds the infant to facilitate standing, walking, etc.
Stimulates gross motor activity	The mother moves infant’s limbs so as to mimic gross-motor behavior
Shifts infant	The mother repositions the infant
Holds object	The mother holds an object
Points to object	The mother points to direct the infant’s attention to something
Offers object	Offering an object by handing it towards the infant
Manipulates object	Use, move, arrange, operate, play, or control a toy or an object to demonstrate how a toy works
Infant-directed speech	If the mother uses coherent words, it is coded as speech.
Affectionate touch	Any sort of touch or behavior that primarily conveys affection.

As described by Sung et al., coders were considered reliable upon having attained an average Cohen’s kappa of 0.60 or above across at least six different observations (Supplementary File S1). To evaluate reliability, a pair of research assistants double-coded 15% of the entire observation. That is, 15% of the approximately 250 observations of mother–infant play together scenes were double coded by two coders. Fifteen percent may seem like a small subset, but current standards for behavioral coding range from 10% to 20% (Murphy et al., 2015; Ladd et al., 2016), and so this behavioral coding meets those standards. Thus, the average kappa score is the mean score of 37 observations. Coding was performed in passes by different coders. First, a coder assigned play frames; then, a group of coders coded infant behaviors, while a different group coded maternal behaviors. Coders specialized in behavior categories such as vocalization, or object play or motor play. The average kappa score across all measures was 0.73. All individual kappa scores were above the cutoff for “substantial” or “acceptable” agreement except for two variables (i.e., holding toy object and passive play—two variables that meet current standards for “moderate” agreement; Danko et al., 2016; Ladd et al., 2016; Marquis et al., 2017; Yao et al., 2017; Gaume et al., 2019). Neither of these variables were significant for the analysis presented in this paper.

We provide full description of these codes in Supplementary Data 1.

### Data Analysis

**FIGURE 1:**
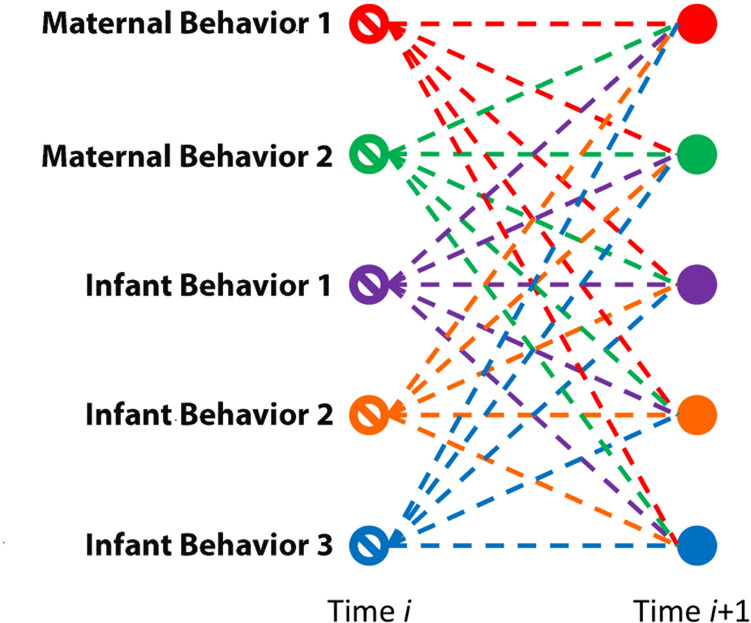
VAR(1). Schematic of relationships modeled by vector autoregression at a lag of one time step.

Vector autoregression modeling and impulse-response forecasting (IRF) are a complementary pair of analyses used to understand relationships in an interactive system. In its simplest case, we might describe the former as providing the basic framework of relationships among potentially causal nodes, aggregated and averaged across time. Here, the nodes are behaviors, either of an infant or of a mother. VAR estimates connection strengths between each pair of nodes, i.e., each pair of behaviors—providing ultimately a web (Figure 1) of relationships summarizing a complex system. It is important to guard always against misconstruing the relationships in a VAR as explicitly causal, but they offer the potential for testing whether two variables are related in an extremely limited but still interesting type of cause, namely, Granger cause, in which the prior changes of one variable are associated with later changes in a second variable above and beyond the second variable’s contributions to its own later changes (see Roebroeck et al., 2005; Xu et al., 2020).

**FIGURE 2:**
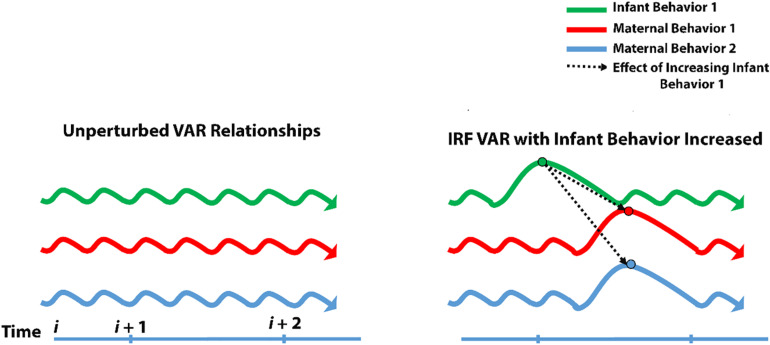
Impulse-response modeling. Schematic of impulse-response modeling and how it serves to test for unique contributions of individual variables engaging in bidirectional relationships.

IRF allows us to reach into this complexity with a mathematical probe and test the effects of manipulating a single node at a time. Taking the web of relationships from VAR, IRF plucks one of the nodes (or more mathematically speaking, simulates an impulse for one of the nodes) and examines what happens to all of the other nodes at a later time (Figure 2).

#### VAR Modeling

We used the R library “vars” to compute VAR and IRF models (Pfaff, 2008), and for the purposes of an accessible tutorial mode aimed at developmental psychologists interested in gender/sex development and in mother-child interactions, we only detail the simplest variant of VAR and IRF. As noted above, more elegant variants of VAR exist where users prefer more theoretical constraints (e.g., Roebroeck et al., 2005). Also, old as the initial VAR modeling strategy is (i.e., almost 40 years since Sims, 1980), a relatively recent update includes, for instance, multi-level modeling into VAR strategies (Bringmann et al., 2013). VAR modeling has traveled unevenly enough across the different subfields to inspire widely different views as to recommended usage. That is to say, far from finding any stable expression of best practices in the psychological field, we find that uses of VAR differ in how researchers adapt VAR to psychological–scientific needs and constraints. We have been fascinated to note that readers of earlier drafts of this manuscript actually diverged quite a bit in their recommendations of best practices: one reader insisted that any comparison of bidirectional effects by gender/sex should require encoding gender/sex as an explicit predictor within fewer models, and another reader recommended fitting a new VAR model for each dyad. We aim to showcase the simplest variant of VAR if only to give developmental psychology the simplest possible entry point to this method for testing bidirectional effects.

##### Background

###### Beyond mean and variance, repeated-measures designs require acknowledging a third component of the linear model

To understand VAR, it helps to go back briefly to the basics of the linear model. This step is not to rehash anyone’s statistical training but to point out a specific foundation of the linear model that many introductory statistics trainings may, for the most admirable pedagogical reasons, have omitted. The linear model comprises three major elements, but many an effective introductory statistics class presents only two, i.e., the mean and the variance (or standard deviation). The third element—called “the autocorrelation function” or just “the autocorrelation”—very often appears only implicitly and, at that, only with zero value in the guise of the “fair coin” that, when flipped, will have equal probabilities of coming up “heads” or “tails” on each flip. To be clear, the “fair coin” metaphor is a statement about memorylessness of events, which amounts to an autocorrelation with all values of zero, but for students first encountering statistics, mean and variance are all we need to steer a semester’s curriculum through analysis of variance and/or ordinary least-squares regression (Tabachnik and Fidell, 2007).

The purpose of all statistics in linear modeling is to account for the variance of a measure *x*. The mean is the sum of measurements divided by the number of measurements (i.e., $\bar{x}$, the sum of all *x*_i_ divided by *N* for *i* = 1,…,*N* where *N* is the sample size), and although linear modeling can focus very much attention on the mean, the mean is usually most interesting insofar as it can vary in a way that informs a hypothesis test. A mean that does not vary is just an intercept on a vertical axis whose value we can take or leave depending on our choice to look at raw or mean-centered values, respectively, of our measure *x*. Variance is, roughly, the average squared difference of each value of *x* (i.e., again *x*_i_ for *i* = 1,…,*N* where *N* is the sample size), and indeed, it appears just as well under another name “mean-squared deviation” (often alongside reference to standard deviation as “root mean-squared deviation” or “root mean square”). Perhaps the best reason to stop with mean and variance is that very many useful statistical procedures only need mean and variance. Very many hypothesis tests aim to find less variance within groups and more variance among the means of those groups. In short, variance is what it is all about (Tabachnik and Fidell, 2007).

The only reason to even consider the autocorrelation is when our measure is not just *x* but, rather, *x*(*t*). That is, autocorrelation only enters into the possibility of definition when *x* is a repeated measure. So, given the need to cover between-groups designs and given the higher costs—not just in data collection but in computation—of repeated-measures designs, we find introductions to statistics widely appealing to the “fair coin” metaphor, the idea that we prefer measures in psychology that do not depend on what we measured before. Obviously, some dependence on the past is ideal or expected: we expect a measure of intelligence to be reliable and reproducible across multiple tests with the same participant, and our ability to fumble for the light switch when we return home in the dark depends on the dimensions of our doorway and electrical circuitry to remain the same across time. However, these examples in the prior sentence are about stability of a mean, and really, the issue of the fair coin failing to resemble its past is about the variance: what we do not want is for repeated measurement to change (i.e., to “vary”) based on previous measurements. So, for instance, our intelligence test should be capable of asking the questions in any sequence, and we do not want significant change in response to any single question because of its following or preceding the any other question–response pair (Tabachnik and Fidell, 2007).

This dependence of *change* based on the past is what autocorrelation is all about. In effect, autocorrelation is all about variance all over again: autocorrelation is the capacity for current variance in a measure to be predicted by past variance. One of the central assumptions of the linear model is that variance is homogeneous across groups but also across time. If you look at autocorrelation too hastily, it could look like autocorrelation breaks that homogeneity-of-variance assumption, and that could be confusing for students just embarking on their statistics training. So, it is not really a mystery that more introductory statistics classes do not teach autocorrelation functions or that autocorrelations are not more familiar to more readers. The fair-coin metaphor suggests that linear statistics actually work best when there are no autocorrelations. That suggestion may be true, but the fair coin is by no means a fact of all repeated measurements and sooner a simplifying assumption that is routinely broken in practice (e.g., Metzler et al., 2014).

###### The autocorrelation function encodes how past values of *x*(*t*) at all possible time lags predict current values of *x*(*t*)

It is central to the success of repeated-measures designs that linear statistical models have the capacity for modeling the departure of our measurements from fair-coin independence across time. This capacity comes from the autocorrelation function as the third and final foundation supporting the linear model (Mandic et al., 2008). We only need this foundation explicitly as noted above when our modeling does not simply address a variable *x* but more specifically a time-varying measure *x*(*t*). These methods apply equally well to spatially varying measures, but given the multiple dimensions of space, the mathematical treatments of autocorrelation usually unfold over the domain of time. *x*(*t*) is called a “repeated measure” or a “time series.” Despite the similar terminology, repeated-measures ANOVA does not use the autocorrelation and only represents the time dependence of repeated measures in terms of an intercept defining each participant’s mean value. We need to look at repeated measures on the order of hundreds or thousands of measurements *x*(*t*). We can distinguish the entire time-series *x*(*t*) from *x*(*i*), the individual *i*th value of *x* at the individual *i*th interval of time [i.e., *x*(*i*) for *i* = 1,…,*N* where *N* is now the total number of equally spaced measurements in time; Box and Jenkins, 1976].

The autocorrelation function is a mathematical way to encode how past values of a time series *x*(*t*) contribute to current values of *x*(*t*). For any time series *x*(*t*) of length *N*, the autocorrelation function is a series of *N* − 1 weights, one weight for each of *N* − 1 possible time lags. Each individual *j*th weight indicates how well, on average, we can predict current values of *x*(*t*) from *x*(*t* − *j*) values. The use of weights in the autocorrelation function is much like the use of weights in regression models: the magnitude and the sign of the weights indicate the size and direction of the average effect of past values on predicted current values.

Weights can be positive or negative or zero, and as far as many introductory statistics curricula go, the weights are all zero for all available time lags. If we look at the fair-coin metaphor, we can lift the veil on the autocorrelation function and see that it had been there the whole time. The best reason more people had not talked about it might have been that all weights equaled zero for all time lags. So, we if had a time series *x*(*t*) composed of 100 coin flips, we would have an autocorrelation function 99 weights representing the contribution of the just-previous coin flip, of the flip before that, and all others leading up to the first coin flip. The autocorrelation function would represent “just previous” as “lag-1” and “the flip before that just-previous flip” as “lag-2,” and the first coin flip would get represented as lag-99. There would be no lag-100 because the last coin flip would be the 100th, and there would not have been a flip 100 flips before that one. However, the fair-coin metaphor entails that each weight for each time lag was zero.

It might be more accurate to say that each weight of a fair coin’s autocorrelation will fail to be significantly different from zero. The purity of an all-exactly-zero autocorrelation in theory begins to flicker from view as soon as we collect an actual sample of 100 real coin flips. The finite size of any series of real coin flip is going to show some non-zero average dependence across flips, but the small-sample limitations is going to entail that the variance of this flip-to-flip dependence will be quite large compared to its average.

We can start to imagine what a set of coin flips might look like for positive time-lagged weights in the autocorrelation function. For instance, if there is only a strong significant positive weight on lag-1, then it would mean that the just-previous flip is a strong predictor of the current flip. So, the first coin flip was “heads” (H), and if we had a positive weight on lag-1, then our coin flips might have shown the following sequence:

HHHHHHHHHHHH….HHHHHH

or if the first coin flip were “tails” (T), the positive weight on lag-1 would entail the following sequence:

TTTTTTTTTTTTTTTT….TTTTTT.

Now, if we kept the same positive lag-1 weight but found a slightly smaller positive weight for this time lag, it would reflect that the flip-to-flip dependence was a little weaker, e.g., yielding less uniform coin-flip series resembling:

HHHHTHHHHHTHHTHHHHHHH….HHHTHH or

THTTTTTTTHTTTHTTTTTTT……TTTTTTTHT

Conversely, a strong negative lag-1 weight would suggest that the just-previous value was a good predictor of the current value, but in the opposite direction. Thus, an idealized coin-flip series with a strong negative lag-1 weight would look like the following:

HTHTHTHTHTHTHTHTHTHTHTHTH or

THTHTHTHTHTHTHTHTHTHTHTHT.

###### Autoregressive (AR) regression models

The autocorrelation can live side by side with any regression model that we estimate for our time-series dependent variables. That is to say, the regression modeling that begins with repeated-measures ANOVA grows seamlessly into the autoregressive modeling that draws on the autocorrelation function. It is the same math for predicting variance in our dependent measures, and it respects the same assumptions about unpredicted variance left over in our residuals. If anything, autoregressive modeling is a bridge between repeated-measures regression model staught introductory statistics training and the linear time-series modeling that supports forecasting in fields like finance.

We can formalize the fair-coin and the lag-1 relationships into a standard regression format. The probability *p* of a fair-coin flip *x*(*t*) coming up “heads” H is 50%. So, *p*(*x* = H) = 50% on average for a fair coin, giving us $\bar{x}$ = 50% if we are collecting coin-flip data, we also expect variability, and we expect the random variability ε to be binomial and, in the long run with larger samples, converging toward Gaussian with variance *s*^2^. Hence, we can write the standard regression formula as follows:

$$\hat{x}\,\left(t\right)=\bar{x}+\varepsilon$$

If we have a coin weighted to yield an average rate of H equaling 75% of the flips, that amounts to updating only the $\bar{x}$ term with a new average value, but the weighted coin might still be “fair” insofar as having no dependence from flip to flip. What this foregoing regression equation entailed is that the time variability of coin flips *x*(*t*) is best approximated as the average outcome plus binomial-to-Gaussian variability. The ε represents the residuals of this regression model, whose variance should remain equal across time and without temporal structure.

Now, if we have a coin with non-zero lag-1 weight in the autocorrelation function, we can update the regression equation. This admission of a significantly non-zero predictive weight from the autocorrelation function becomes known as “autoregression” (AR). For what it is worth, we know to estimate an autoregression model when the residuals ε from the above model show temporal structure (sometimes called “serial correlations,” indicating that there are statistical relationships across the sequence of measurements). If the residuals ε show temporal structure, then it means that the model is poorly specified. Residuals ε with temporal structure require that regression modeling include a weight from the autocorrelation function. That inclusion looks like the following:

x^(t)=x¯+Bx1(t-1)+ε,AR(1)

where *x*(*t* − 1) represents the average of lag-1 values of *x*(*t*), B_1_ represents the autocorrelation weight for lag-1 on predictions of current values of *x*(*t*). Note that ε_AR(__1__)_ is a new residual term that differs from ε because we have fit a new regression model. “AR(1)” is mathematical notation that means “autoregression using lag-1 autocorrelation weights,” with “AR” abbreviating “autoregression” and “1” equaling the maximum lag used. The B_1_ weight is directly related to the autocorrelation weight for lag-1. If B_1_ is positive, then we would have series like “HHHHHHH…HHH” or “TTTTTTT…TTTT.” If B_1_ is negative, then the just previous coin flip should predict the opposite outcome for the current flip, e.g., “HTHTHTHTHT.”

When would we ever use autoregression? The statistical housekeeping here requires attention to the residuals of each of these models. We should only have fit the autoregressive model if the residuals ε had temporal structure. According to the linear model, ε is assumed to arise from the sum of many independent random variables, and it is the independence of these component random variables that should guarantee the homogeneity of variance, and so any structure in ε suggests that there are deterministic relationships to the dependent measure *x*(*t*) hiding in the ε term. So, because the central limit theory assumes that measures are the sum of deterministic components and non-deterministic components, the variance of ε should be the sum of two variances: variance due to *x*(*t* − 1) and variance due to ε_AR__(__1__)_. Ideally, ε_AR__1_ should differ from ε in two respects: ε_AR(__1__)_ should have smaller variance than ε, and ε_AR__(__1__)_ should have weaker evidence of temporal structure than ε because the AR model will have, by definition, controlled for lagged values of *x*(*t*).

How much autoregression can we fit into our models? The answer to this question requires attention to our residuals again. If ε_AR(__1__)_ turns out to have temporal structure remaining, then regression modeling may benefit from further autoregressive terms, that is, from using any further weights from the autocorrelation for more time lags than just lag-1. Effectively, regression modeling has access to all *N* − 1 time lags evaluated in the autocorrelation function. We can generalize the autoregressive formalism to all time lags that we might use in our regression model as follows:

x^⁢(t)=x¯+B⁢x1⁢(t-1)+B⁢x2⁢(t-2)+…+B⁢xp-1(t-p+1)+Bxp(t-p)+ε,AR(p)

yielding a generic form for all autoregressive models AR(*p*) where *p* is equal to the maximum time lag incorporated into the model. The same wisdom holds for autoregressive terms as for higher-order interactions: if they do not improve model fit, we do not include them (e.g., Allison, 1977).

When does the autoregression stop? In theory, because there are *N* − 1 time lags available from the autocorrelation function, an AR model could include up to *p* = *N* − 1 autoregressive predictors. However, in practice, it is extremely unlikely to get any but short-lag models. Recall that each weight for each *p*th time lag represents an average contribution across that given time lag, and the average weight is only going to be as informative as the sample size of lag-*p* relationships increases in the measured time series. In a 100-flip time series of coin flips, there is only one lag-99 relationship in evidence, i.e., that between the 1st and the 100th coin flip. Meanwhile, there are 99 instances of a lag-1 relationship in the same series. Hence, there will be a much more stable estimate of lag-1 effects than of the lag-99 effect. For this reason, autoregressive models usually pertain to only short lags (e.g., lags 1 through 5; Wagenmakers et al., 2004). Long-range relationship between lagged and current values appears in linear modeling as an exceptional case (e.g., fractional integration; Granger and Joyeux, 1980) or as a symptom of non-stationarity (Box and Jenkins, 1976). Neither case is required to understand AR or VAR models, but both cases warrant corresponding steps to make them more tractable for regression-modeling purposes. We note them only to help distinguish the scope of AR and of VAR. It is extremely likely that residuals for the AR(*p*) model will stop exhibiting temporal structure at *p* much less than *N* − 1. Recall that we only fit new autoregressive terms if the residuals ε have temporal structure, and so as soon as residuals ε_AR(_*_p_*_)_ show no temporal structure, then we will have satisfied the long-standing assumption that variance should be homogeneous across time. Once the residuals exhibit the signs of no-structure homogeneity across time, then we can conclude the AR model.

###### The purpose and the scope of vector autoregressive (VAR) regression models

The econometrician Sims (1980) developed VAR modeling quickly on the heels of innovative developments in time-series modeling such as by Box and Jenkins (1976). The motivation to develop VAR was that macroeconomics posed two major challenges to statistical modeling. First, economics on the macro-scale of whole industries, nations, and multi-national trade organizations could never draw on truly experimental work with random assignment to treatment groups. Second, each measurable time series in macroeconomics was tangled up in many others. For instance, gross domestic product, imports, exports, wages, unemployment, physical plant processes, and consumer spending all influenced one another. As a consequence, many regression models that presumed to name a predictor variable, to name an outcome variable, and to estimate the effect of one on another could only make the most hedging, contingent conclusions about underlying relationships. Not only was macroeconomics stuck doing correlational research, but whereas correlational research is burdened with an uncertainty as to which variable causes which, macroeconomists such as Sims were fairly sure that multiple variables might just be both cause and effect amid a thicket of interlocking relationships.

Vector autoregression modeling presumes to apply the same logic as motivated AR modeling of time series *x*(*t*), but it broadens the class of autoregressive predictors to include lagged values not just of *x*(*t*) but also of past values of other time series. The same logic means that VAR respects the same assumptions of residuals that are homogeneous in variance and so free of structure across time. VAR is only valid for time series with short-lag autocorrelations, and non-stationarity and fractional integration require explicit extensions of the basic VAR framework. As it applies to multiple time series, VAR requires that the time series are cotemporaneous. That is to say, because we are going to be using the same *p*-lagged values of multiple time series, we need the time series to begin at the same time and to progress with the same time intervals (Lutkepohl, 2005).

As linear models go, VAR is agnostic to the underlying network of relationships among all potentially causal and potentially effect variables. Except for the assumptions of homogeneous variance and of short-lag relationships, VAR allows the capacity to explore all possible relationships. Just as there are VAR elaborations to suit non-stationarity and fractional integration, there is a less exploratory variant of VAR called “structural VAR” (SVAR) that allows prior delimiting of available autoregressive relationships among the variables. However, because we come to our questions about gender/sex relatively skeptical about theoretical preconceptions, we do not present SVAR here. The drawback of taking such a shotgun approach to modeling is that the results are copious (Lutkepohl, 2005).

Vector autoregression generalizes the same autoregressive framework to a multivariate (i.e., multiple dependent variable) case for which, rather than predicting the current value of a single time series *x*(*t*), it predicts current values of a whole system of multiple time series *x*_1_(*t*),*x*_2_(*t*),…*x_m–__1_*(*t*),*x*_m_(*t*), where *m* is the number of time series in the system. Although it is possible to treat some of the variables as exogenous, i.e., as influencing other variables without responding to any other variable, the strength of VAR modeling lies in the capacity to treat variables in this *m*-dimensional system as endogenous, i.e., as both influencing and responding to other variables. Hence, for every endogenous variable *x*_j_(*t*) for *j* ≤ *m*, VAR models regression coefficients for all *p* past values of all *m* variables, i.e., *x*_1_(*t* − *p*),*x*_1_(*t* – *p* + 1),…*x*_1_(*t* − 2),*x*_1_(*t* − 1), *x*_2_(*t* − *p*),*x*_2_(*t* − *p* + 1),…*x*_2_(*t* − 2),*x*_2_(*t* − 1),*x_m–__1_*(*t* − *p*),*x_m–__1_*(*t* − *p* + 1),…*x_m–__1_*(*t* − 2),*x_m–__1_*(*t* − 1), *x*_m_(*t* − *p*),*x*_m_(*t* − *p* + 1),…*x*_m_(*t* − 2),*x*_m_(*t* − 1). Whereas AR modeling only allowed testing the effect of past values of a variable on current values of itself, VAR modeling accomplishes the same as AR but, in addition, simultaneously allows testing the effects of past values of many other variables on current values of itself (Lutkepohl, 2005). To illustrate with a more compact case, if we have two time series *x*_1_(*t*) and *x*_2_(*t*), then the VAR(1) model would look like:

x^(t)1=x¯+1Bx1(t-1)1+Bx2(t-1)2+ε)1VAR(1

and

x^(t)2=x¯+2Bx3(t-1)2+Bx4(t-1)1+ε.2VAR(1)

Hence, whereas AR(1) models only feature one instance of *x*(*t* − 1), now, we see that the same lagged value, e.g., *x*_1_(*t* − 1), appears twice in the two-time-series VAR model and so prompts modeling two separate coefficients, e.g., B_1_ and B_4_, the former and the latter contributing to predictions $\hat{x}$_1_(*t*) and $\hat{x}$_2_(*t*), respectively (Lutkepohl, 2005).

It is noteworthy that each equation has its own residual term. Model stability depends on whether the residuals are identically distributed and whether they are independently distributed across time—that is, again, we have to satisfy those perennial assumptions of homogeneous variance and of no temporal structure. Just as failure of these assumptions led us above to include another lagged value, if a VAR model fails either of these assumptions, it is important to incrementally increase the parameter lag *p* until these requirements of the residuals are met. Otherwise, the VAR model would not be stable and would not provide a judicious model of short-lag effects. Testing these conditions requires multivariate tests for heteroscedasticity (i.e., failures of homogeneous variance) and for serial correlations (i.e., failures of independence across time). Both of these tests are available with the R library “vars” (see Supplementary Data 2 for example script).

###### Limitations of VAR

Despite being an elegant means for investigating short-lag effects of multiple variables on each other, VAR suffers from all the same limitations of correlational analysis. Although impulse-response functions (see section “Impulse-Response Modeling”) address some of the question of directionality (i.e., which of the related variables influences which others), the fact remains that poor selection of the variable or of the proper sample size is always a threat to interpretation. There are likely more variables and more measurements that would inform the conclusions. As ever, correlation does not entail causation. The specific strength of VAR is that it can establish correlation of one variable’s increases with another variable’s later changes. Beyond that strength, VAR is prone to spurious correlations as any other regression model.

Spurious false positives are a problem for all correlational analyses, but no matter whether correlation does not entail causation, causation should at some point produce correlation. So, perhaps a more important point is to delimit the types of causal relationships that might register a true significant effect in VAR. In this light, the more specific limitation of VAR is that it can only estimate relationships between variables that exist across short lags. That is, it can only estimate relationships between variables that unfold across the briefest time scales. For instance, it is possible that changes in one variable only have effects on another variable that appear at a very long delay. A second possibility is that the actually causal interactions between variables unfold across multiple time scales at once. Both sorts of causal relationships are expected in developmental psychology where we cannot easily assume that all variables change in the same way (Adolph and Robinson, 2011). VAR would be insensitive to any causal relationships, and to get at long-range or multi-scale relationships, we would need the vector error correction (Lutkepohl, 2005) and vector fractional integration models (Podobnik and Stanley, 2008). For present purposes, we hope that it is a worthwhile step to introduce VAR as a first constructive step.

##### Present application of VAR

We ran four lag-1 VAR models across all infants using the month-by-month frequency of variables described in Table 1, that is, VAR models with lag *p* as noted in the previous paragraph. We only ran lag-1 VAR models because lag-1 was sufficient to generate stable models according to the requirement of identically and temporally independently distributed residuals. This modeling strategy allowed us to test whether different maternal and infant behaviors influenced each other, controlling for all relationships of all previous values of variables with all current values of the variables. Two models used series encoding duration in seconds, and two models used series encoding frequency as number of events. One of each model type pertained to male infants or their mothers, and the other of each model type pertained to female infants or their mothers.

#### Impulse-Response Modeling

##### Conceptual motivation for using impulse-response functions (IRFs)

Impulse-response functions use VAR models to generate a prediction of each variable’s unique effect on another variable in the same system of equations. There is no principled reason why the regression coefficients are closed to direct interpretation, but various mathematical treatments of VAR suggest a general distaste for attempting to interpret the raw coefficients. Indeed, the problem is not that the coefficients are not trustworthy, but if you have 26 variables each participating in the VAR model, the simplest case of *p* = 1 requires reading (26 lagged values × 26 equations =) 676 coefficients. What the impulse-response functions do is iterate predicted values forward into time based on time-lag coefficients. This method generalizes the idea of Granger cause (see section “Data Analysis”), projecting predicted values of each variable over several time steps into the future in response to unique variables. In the econometrics parlance, the impulse-response function simulates a “shock” from each individual variable and then uses the VAR model coefficients to project how that shock from one variable prompts later responses from predicted values of the other variables. Granger cause is not all of cause, and perhaps Granger cause is better understood as prediction. However, the benefit of Granger cause and impulse-response functions is that they model relationships between variables in which one variable’s increase is prior to the predicted values of another variable (Lutkepohl, 2005).

##### IRFs use residuals to simulate instantaneous shocks from unique variables

Impulse-response functions leverage both the VAR coefficients and the residuals. The VAR coefficients encode average time-lagged relationships. We can think of the set of VAR coefficients as a skeleton of linkages composing the network of variables. This skeleton holds on average and none of the weights are prior to one another. So, they are not sufficient to make predictions about unique effects from one variable on another. On the other hand, the residuals of the VAR model describe how the measured time series vary above and beyond their average. So, the residuals are where impulse-response functions take hold. Impulse-response functions aim to simulate the effect of a “shock,” i.e., a drastic increase in a unique variable. For instance, in a two-variable VAR, modeling the effect of prior *x*_1_ on later *x*_2_ involves artificially increasing the residual term for ε_1__VAR(_*_p_*_)_ for one-and-only-one time step while leaving the residual term ε_2__VAR(__1__)_ unchanged. The question for the impulse-response function is whether this instantaneous increase in the residuals in one variable has a later effect on the other variable (Lutkepohl, 2005).

##### Enforcing uniqueness of simulated shocks requires orthogonalizing residuals

The difficulty in estimating an impulse-response function to portray unique effects is that the residuals in any VAR(*p*) model are correlated with one another. Correlation of residuals is an expectable fact of causally related variables. Unique effects are what scientists want to know about. So, to make up the difference between inevitable correlation and desired uniqueness, the common resolution is to orthogonalize the matrix of residuals (i.e., a matrix of *m* columns and *N* rows, where *m* is the number of regression equations and *N* is the length of the time series). Generally, VAR-IRF procedures employ Cholesky decomposition to accomplish this orthogonalization. Once orthogonalized, it is possible for the statistician to reach into the residuals and to produce a “shock” by adding a standard error at one and only one time step. That standard error increase will increase the predicted value for that one variable at that one time step, but then, at the next time step, the increase in that one variable will have spread through the entire system of equations. So, orthogonalization of residuals allows simulation of how unique variables have unique effects on later changes in other variables (Lutkepohl, 2005).

##### IRFs shed light on directionality of effects between interactive variables

The impulse-response function is thus a very important attempt to resolve the directionality problem of correlational research. Whereas pairwise correlation only expresses association and does not indicate which of the two variables might influence the other, impulse-response functions provide a better way to express which variable could possibly influence which other variable. It is certainly true that there remains a “third-variable problem” in VAR because researchers could always have failed to include all of the relevant variables. However, with the capacity to model uniqueness and priority of one variable’s earlier change improving prediction of another’s later change, VAR has been an important step forward in grasping for any glimpse of causal relationships among many interacting, time-varying measurements (Lutkepohl, 2005).

##### Computing average values of IRFs

Impulse-response function modeling simulates an “impulse” by increasing each of the *m* variables at a time by a standard error, and lastly, it allows this brief increase to propagate through the vector-autoregressive system’s regression coefficients over many time steps into the future. The default number of time steps in the R library “vars” is 10. Responses to impulses decay to zero, but in the short range of 1 to 10 or so steps forward in time, impulse-response modeling can show that an increase in one variable can promote either an increase or a decrease in another. Hence, IRF allows us to test the unique effects of each variable on later values of other variables. These average values predicted by charting the effect of a unique-variable shock can vary across time and across different variables. Any given variable can respond more or less to a given shock, and some variable’s shocks can have stronger or weaker effects. Hence, IRFs do not simply provide a binary signal of future effects of earlier shocks. Given the abundance of effects in the present paper, we will collapse most of these IRF results into binary information (e.g., there was a significant later response, or there was not), and we will indicate the direction of this later response (e.g., the later response was positive, or it was negative; Lutkepohl, 2005).

##### Computing 95% confidence intervals for IRFs

The VAR coefficients provide means for calculating the average value of later responses to earlier shocks, but identifying statistical significance of non-zero responses requires a 95% confidence interval for each time step into the model-predicted future. The standard way to estimate the confidence interval for IRFs is to bootstrap multiple new model predictions from the VAR. This bootstrapping procedure involves resampling the residuals with replacement, that is, taking a randomized subset of the actual residuals and randomly reshuffling their order across time. Reshuffling the residuals of the VAR model 100 times can produce slightly different model-predicted values across time. These 100 different model predictions generate a 95% confidence interval, and significant forecast effects are indicated when the bootstrap-generated 95% confidence interval does not include zero (Lutkepohl, 2005).

##### Fine print for conservative estimation of IRFs for any given (or all) variables

Producing conservative estimates of any given variable’s unique effects on the other variables in a VAR model requires entering that variable last (i.e., as the rightmost column) in the matrix submitted to the VAR-estimating function. For each of the four VAR models noted above, we ran VAR modeling with 26 different orderings of the variables to ensure that we could report only the most conservative estimates of each variable’s impulse effects on all other variables. Each of those 26 models had a different variable occupying the rightmost column in the matrix submitted to the R script for VAR estimation. This resorting might strike a newcomer as unduly cumbersome, but it is necessary in order to avoid overstating a given variable’s impact by leaving it in a position other than rightmost-in-the-matrix.

The reason that order of variable entry matters has to do with the iterative nature of Cholesky decomposition for orthogonalizing the residuals. Order of variable entry will not change VAR coefficients in the least. VAR is symmetric across variable entry: coefficients estimated for [*x*_1_,*x*_2_] are exactly the same as those coefficients estimated for [*x*_2_,*x*_1_]. It is conceptually similar to partial correlations between two variables. Partial correlations between *x* and *y* are often less than simple pairwise correlations because partial correlations begin by taking account of correlations between *x* and a third variable *z*. Similarly, the first column in the orthogonalization matrix produced by a Cholesky decomposition includes the standard deviation *s*_1_ for the first-entered variable’s residuals ε_1__VAR(_*_p_*_)_ in the top row, and all other rows in that first column contain the covariance terms (covariance between first variable and each other variable) divided by *s*_1_. Then, as the Cholesky decomposition iterates, it fills each new column of the orthogonalization matrix and is calculated by subtracting or dividing the previous column’s entries (e.g., for *x*_1_) from the next variable’s residual [e.g., ε_2__VAR(_*_p_*_)_] standard deviation and covariance terms. Hence, the structure of the residuals appear untempered for ε_1__VAR(_*_p_*_)_, but each subsequent set of residuals [e.g., ε_2__VAR(_*_p_*_)_, ε_3__VAR(_*_p_*_)_, …ε_m–__1__VAR(_*_p_*_)_, ε_m VAR(_*_p_*_)_] is represented in the orthogonalization matrix with progressively more of the preceding variables tempering their variance contributions. So, the last entered variable’s residuals ε_m VAR(_*_p_*_)_ participate in impulse-response functions only after the variance of all other residual terms has been controlled for, and this iterative tempering makes the IRF test of later responses to prior impulses most rigorous for impulses produced by the last-entered variable (see Supplementary Data 2 for example in R script).

## Results and Discussion

**FIGURE 3:**
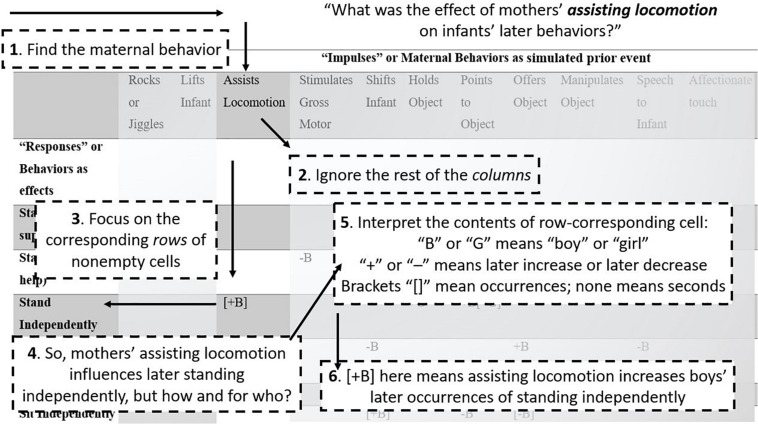
How to read the tables. A step-by-step guide to reading Tables 2, 3, guiding readers through Table 3 as an example, following the question “What was the effect of mothers’ assisting locomotion on infants’ later behaviors?”

**FIGURE 4:**
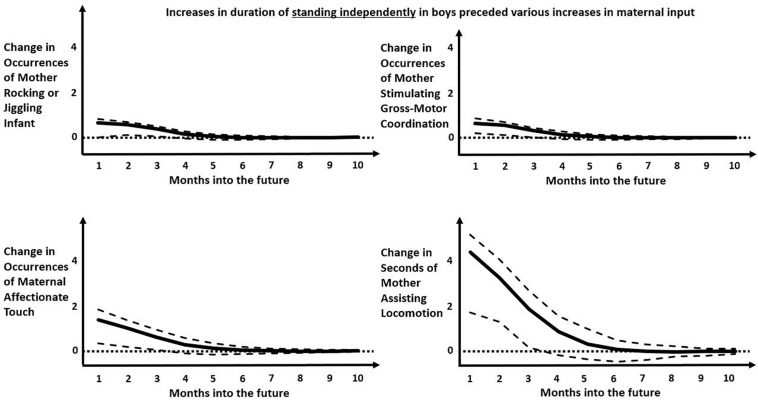
Impulse-response functions modeling the predicted later increase in various maternal behaviors following an increase in occurrences of boys standing independently. Solid black curve represents the average IRF values over months into the future. Dashed lines represent the upper and lower bounds of the 95% confidence interval. Dotted lines represent zero-change. Predicted occurrence of “mother rocking or jiggling infant” increased 2–3 months into the future (top left). Predicted occurrence of “mother stimulating gross-motor coordination” increased 1–2 months into the future (top right). Predicted occurrences of “maternal affectionate touch” increased 1–2 months into the future (bottom left). Predicted occurrences of “mother assisting locomotion” increased 1–3 months into the future. Note that, although all predicted later changes are positive and significant over the first few months, the predicted later effects in the top two panels are much smaller than the predicted later effects in the bottom two panels.

**FIGURE 5:**
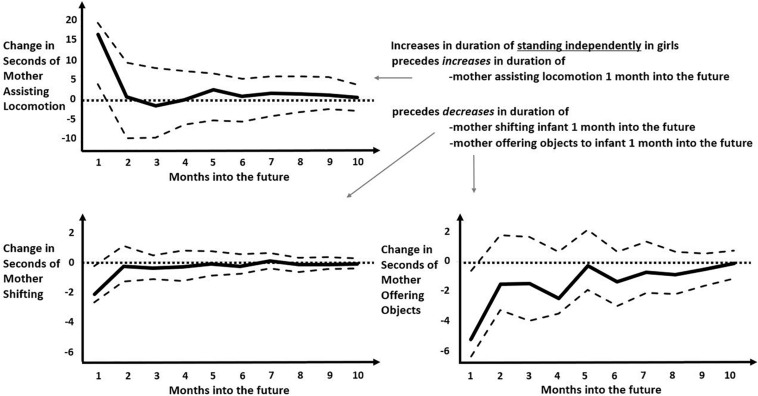
Impulse-response functions modeling the predicted later changes in various maternal behaviors following an increase in durations of girls standing independently. Solid black curve represents the average IRF values over months into the future. Dashed lines represent the upper and lower bounds of the 95% confidence interval. Dotted lines represent zero-change. Unlike in Figure 4, all predicted later responses are relatively small and all persist only 1 month into the future, with small increase in predicted duration of “mother assisting locomotion” (top left) and small decreases in predicted duration of “mother shifting” or “mother offering objects” (bottom left and bottom right, respectively).

**FIGURE 6:**
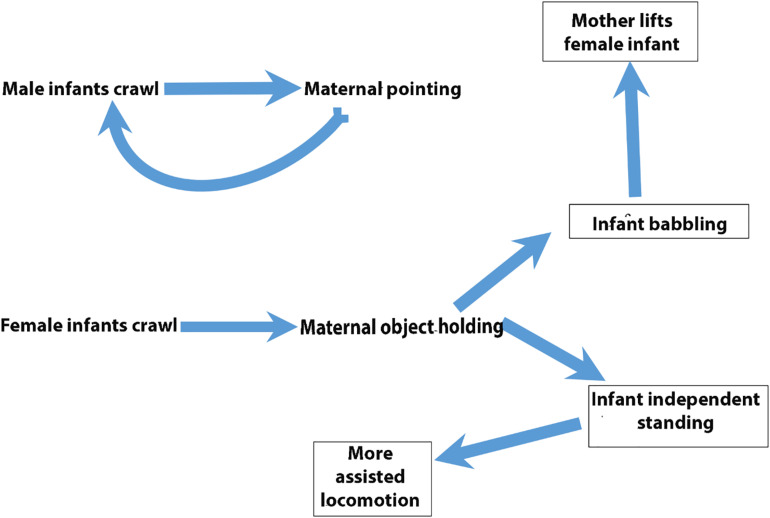
Relationships among behavioral codes. Cartoon of relationships among behavioral codes distinguished by vector-autoregressive and impulse-response modeling.

Tables 2, 3 present the significant (at *p* < 0.05) results of IRF in two directions. Table 2 shows impulse-response results indicating the responses of infant behaviors to previous maternal behaviors. Table 3 shows impulse-response results indicating the responses of maternal behaviors to previous infant behaviors. To help orient readers through these tables, we have included Figure 3 to guide the eye through all of the detail. Figures 4, 5 depict example IRF curves with average effects and 95% confidence intervals to illustrate effects reported in Section “What Infant Behaviors Elicit Changes in Mothers’ Behavior that Differ by Gender/Sex of Infant?” Figure 6 offers a pictorial summary. These results from the IRFs reflect the output of four separate applications of VAR: two for dyads including girl infants (one for durations and the other for occurrences of each behavior code) and two for dyads including boy infants (one for durations and the other for occurrences of each behavior code). Raw data are available on request.

**TABLE 2:** Matrix indicating presence/absence (B or G/blank) and direction of change (+ or −) in boy or girl infant (B or G) behaviors (rows) in response to increases of maternal behaviors (columns) 1 to 3 months beforehand: Relationships between durations in seconds appear outside of brackets; relationships between numbers of occurrences appear inside brackets.

	*“Impulses” or Maternal Behaviors as simulated prior events*										
	Rocks/Jiggles	Lifts Infant	Assists Locomotion	Stimulates Gross Motor	Shifts Infant	Holds Object	Points to Object	Offers Object	Manipulates Object	Speech to Infant	Affectionate Touch
*“Responses” or Behaviors as effects*											
Stand (Object support)							+G				
Stand (Mother help)				−B			−G[−G]	[+G]			+B
Stand Independently			[+B]			+G	+B[+G]				
Sit (Object support)		+B			−B			+B		−B	
Sit (Mother help)							[−G]		[−B]		−B
Sit Independently					[+B]		−B	[−B]			
Lie (All)				[+B]					−B	[−G]	
Lie Still				[+B]			+B		−B	[−G]	+B
Babble						+G[+G]					
Cry	-G										
Reach							+G[-B]	[−G]		−G	[−G]
Crawl					[+B]		[+B]				
Play (Passive)				[+B]		+B				[+B]	
Play (Motor-Social)				+G[+B]			[−G]				
Play (Object)	[+B]	−B		[+B]						[+B]	

**TABLE 3:** Matrix indicating responses of maternal behaviors (direction of change (+ or −) in mother of a boy or a girl infant (B or G) in response to increases in previous infant behaviors 1 to 3 months beforehand.

	*“Impulses” or Infant Behaviors as simulated prior events*														
	Lie_All_	Lie_Still_	Reach	Crawl	Sit_M_	Sit_O_	Sit_I_	Stand_M_	Stand_O_	Stand_I_	Babble	Cry	Play_M–S_	Play_Obj_	Play_Pass_
“Responses” or Behaviors as Effects															
Rocks/Jiggles Infant				[−G]		+ G		−B		[ + B]					
Lifts Infant											[ + G]		+ G		
Assist Locomotion				[−G]		+ G	[−B]			+ G[+B]					
Stimulate Gross Motor	+ B	−B		[−G]					[−G]	[ + B]				[ + B]	[−B]
Shifts Infant		−G								−G					
Holds Object	[ + B]	[−B]		+ G[−B]	[−B]	[−G]	[ + B]		[ + B]			+ G		−B	
Points to Object				+ B			+B								
Offers Object		−G		−G		−G	−G		−G	−G[−G]					
Manipulates Object						−G				−B[ + B]					
Speech to Infant									−B						
Affectionate Touch				[−G]						[ + B]					

*Relationships between durations inseconds appear outside of brackets; relationships between numbers of occurrences appear inside brackets.*

### Mothers’ Behaviors Shape Infants’ Behavior

#### Extending a Known Effect From the Literature on Language Development to Motor Development: VAR Confirms That Pointing Influences Later Infant Motor Behaviors

Infant behaviors showed significant changes subsequent to increases in maternal pointing (Column 7; Table 2; “Points to Object”). When mothers point to an object, the act appears to increase the time (duration) girl infants spend reaching but decreases the number of occasions (frequency) boy infants reach. However, both boy and girl infants show an increase in duration and occurrence of standing independently. This result stimulated us to return to our original data set to see if there were gender/sex-related differences in the frequency or duration of maternal pointing.

#### Disconfirming a Likely Extrapolation From the Literature: VAR Shows That Stimulation of Gross-Motor Coordination Encourages Boys to Lie Down/Still

Gross motor stimulation (Column 4; Table 2) elicited more motor-social play in both mother–son and mother–daughter dyads. Beyond this similarity, however, the effects of gross-motor stimulation diverged for male compared to female infants. Gross-motor stimulation increased passive play and object play frames for mother–son dyads but not mother–daughter dyads. Rather than encouraging more physical activity later on in boys, increasing maternal stimulation of gross-motor coordination actually increased the number of times boys lay down (all sub-codes) and lay still. Given the presumption that motor stimulation strengthens muscles and neuro-muscular connections, a less surprising finding was that gross-motor stimulation reduced the time boy infants spent standing with maternal support.

### What Infant Behaviors Elicit Changes in Mothers’ Behavior That Differ by Gender/Sex of Infant?

#### Opening Up New Avenues for Research: Standing Independently and Crawling Show Many Significant Relationships With Later Infant Behaviors, and Mothers Respond More to Boys and Less to Girls

The most populated columns in Table 3 are for later effects of standing independently and crawling, indicating that these infant behaviors are most likely to change later maternal behaviors. For the most part, infant crawling or standing independently accrue later increases in maternal involvement for boys but, in a novel result, appeared to decrease later maternal involvement for girls. When male infants stand independently, that change predicts more numerous later occurrences of mothers rocking/jiggling, assisting locomotion, stimulating gross-motor coordination, manipulating objects (though spending fewer seconds in this behavior), and affectionately touching infants (Figure 4). In contrast, the only increased later maternal involvement for female infants’ standing independently was time spent assisting locomotion. Otherwise, girls’ standing independently predicted significantly less duration of maternal shifting and less duration (and fewer occurrences) of mothers offering objects later on (Figure 5). As can be seen, the predicted later changes are relatively small for impulse of standing independently by girls, but the predicted later changes in response to an impulse of standing independently by boys shows a range of different sized effects.

Girls’ crawling reduced the number of later occurrences of rocking/jiggling, assisting locomotion, stimulating gross-motor coordination, and affectionate touching of the infant. Girls’ crawling predicted later increases of the time that mothers held an object (toy) but later decreases of the time that mothers spent offering an object (toy). Boys’ crawling has fewer effects on maternal behaviors: mothers spent more time pointing and holding toy objects on fewer occasions. Similar effects appeared in sitting: Boys’ sitting independently leads mothers to assist locomotion less but to hold toy objects and point to toy objects more. Girls sitting independently only predicts that mothers will later spend less time offering objects.

The foregoing remarks refer only to a small portion of the existing results. We have included Tables 2, 3 in their entirety as well as Figure 3 offering pointers for further reading of these tables so as to offer the reader an opportunity to see the results in their entirety. Readers hoping to check their understanding of the remainder of the results not explicitly discussed can consult a complete list of all significant effects in online Supplementary material (Supplementary Data 3, 4). A big data set presents many analytic challenges. VAR and subsequent IRF offer a way to let the data speak to bidirectional relationships and bring into relief, through traditional filters of significance testing, those strongest relationships that interested researchers may probe with subsequent experimental test. Figure 6 cartoons the reviewed findings.

## Conclusion

We demonstrate that VAR serves the known needs for hypothesis testing but brings the traditional rigor for testing hypotheses to the space of bidirectional relationships: it can expand known effects, disconfirm likely hypotheses suggested by the literature (e.g., that gross-motor stimulation encourages greater physical activity in boys), and open up new avenues for research that prior literature and modeling have not proposed or explored. As in the introductory remarks, we emphasize that these findings serve as examples of findings that VAR modeling could potentially generate and that might answer old questions and unearth new questions for future research. We address the results of latter type in our remaining remarks.

Because gender/sex-related differences in activity levels and active play styles are widely reported (Campbell and Eaton, 1998; Lillard, 2015), we originally developed the motor codes used here with the hypothesis that mother–infant dyadic interactions would contain observable antecedents to later gender/sex-related differences. Thus, we were not surprised to find mothers respond to boys’ physical activities with greater physical stimulation and affectionate touch. However, we did not anticipate the current study’s findings that motor activities from girls would lead to a decrease (as opposed to no effect) in gross motor stimulation, rocking and jiggling, interactive object play, and affectionate touch. These kinds of results add weight to the claim that rather than being a piecewise process starting with biology and expanding via socialization, gender/sex differences in play and physical activity are emergent, interactive properties produced through the day-to-day physical interactions between infant and caregiver (Fausto-Sterling et al., 2015).

In the current study, the forecasted effects of simulated impulses are on the order of months rather than minutes, days, or years. However, our results can instruct us on how to design a study that could begin to expose these steps on a more fine-tuned time scale following the caution laid out by Adolph and Robinson (2011) of the need for time dense sampling in developmental studies. Nor is it unusual for longitudinal studies to sample on the time scale of months and even years (Beebe et al., 2010; Feldman, 2015). Our results also lead to hypotheses about developmental dynamics or chains of events. For example, as cartooned in Figure 6, we found a feedback loop in which male infant crawling might lead at some later date to more maternal pointing, and more maternal pointing might lead to more male infant crawling. For female infants, we uncovered a more open-ended set of events. Female infant crawling results later in an increase in maternal object holding, which in turn leads to increases in independent standing and babbling for girls. Increased independent standing for girls appears to precede more maternally assisted locomotion, but a decrease in the frequency with which mothers offer objects to their daughters.

We did not expect looped versus more open-ended feedback for mother–son versus mother–daughter dyads, but this finding might add to existing understandings of dynamic social processes during gender/sex differentiation. Children can recognize gender/sex (e.g., distinguish male from female faces) in their world as early as 12 months. But as we show here (and as others show in a wide-ranging literature), the patterns of handling, feedback, and touch–response–touch behavioral loops and chains differ in mother or father–son and mother or father–daughter dyads (Feldman et al., 2006, 2010; Feldman and Eidelman, 2009; Feldman, 2015). As infants incorporate a bodily sense of self and develop response expectations from the adults with whom they interact, they also observe gender/sex-stereotyped behaviors of others in their world, producing behaviors and beliefs varying in the fierceness with which they are held (Zosuls et al., 2009; Ruble et al., 2010).

The application of VAR-IRF analyses to a detailed, longitudinal data set opens a novel window onto the thicket of causal relationships supporting gender/sex development and differentiation. No matter the elegance of the statistical modeling strategy, it is of course incumbent on critical consumers to acknowledge that VAR- and IRF-based estimates indicate relationships that, much like pairwise correlation, cannot be equated with causality. However, it is important to evaluate our suspicions as to bidirectional relationships where we suspect that one variable may influence future values of another variable. Our results provide a promising existence proof for testing multimodal, bidirectional relationships in the gender/sex-dependent interactions of children and their caretakers. We do not intend for this existence proof to stand for explanations of each of the results, but we expect that such modeling could prove useful for developmental research. We hope that the results serve to give substance to what might otherwise have been a dry mathematical tutorial. Future research in the development of gender/sex identity and expression might build on these early results using VAR-IRF analyses, with elaborations of particular early dyadic interactions between infant and caregiver, and offer specific focus on how infants born into a highly gender/sex-designated culture learn to internalize individual identities.

## Data Availability Statement

The datasets generated for this study are available on request to the corresponding author.

## Ethics Statement

The studies involving human participants were reviewed and approved by Brown University Institutional Review Board. Written informed consent to participate in this study was provided by the participants’ legal guardian/next of kin.

## Author Contributions

EE contributed to planning analysis, organizing behavioral coding data, began data analysis, as well as contributed to composition of the manuscript. NC contributed to data analysis and to the composition of the manuscript. DK-S contributed to planning analysis, organizing behavioral coding data, and composition of the manuscript. AF-S contributed to planning analysis and composition of the manuscript. All authors contributed to the article and approved the submitted version.

## Conflict of Interest

The authors declare that the research was conducted in the absence of any commercial or financial relationships that could be construed as a potential conflict of interest.
