# Supplementary material for: Using Vector Autoregression Modeling to Reveal Bidirectional Relationships in Gender/Sex-Related Interactions in Mother–Infant Dyads
Source: Front Psychol. 2020 Aug 5;11:1507. doi: 10.3389/fpsyg.2020.01507 (PMC7419485; doi:10.3389/fpsyg.2020.01507)
Supplement: Supplementary file 1 [file Data_Sheet_1.docx]

Supplementary Material

Using vector-autoregressive modeling to reveal bidirectional relationships in sex-related interactions in mother infant dyads

**Elizabeth G. Eason, Nicole S. Carver, Damian G. Kelty-Stephen*, and Anne Fausto-Sterling**

*** Correspondence:** Damian G. Kelty-Stephen, foovian@gmail.com

**Supplementary Data 1.** **Description of our complete coding scheme.**

**Infant Activity**

*The category of infant activity is designed to capture the postural and locomotive status and gross motor limb movement of the infant.* *Big five locomotion or activities (Lying, Sit, Crawl, Stand, Walk) reflect whole body activity or locomotion, while the remaining three activities (Reaching, Bat/Banging, Pointing) reflect upper body, that is, shoulder, arm, elbow, and hand movements.*

*Pauses in active states which last less than 1.5 seconds were not coded as interruptions of the active state. (e.g. An infant who is crawling for 4 seconds, then pauses in crawling position for 1 second, then continues to crawl for another 5 seconds will be coded as having crawled for the entire 10 seconds.) In contrast, a short burst of activity was coded regardless of duration. When an infant is transitioning from one locomotion/position (e.g. sitting to walking) to another, coding switches over when the new activity begins (e.g. first step), not when the old activity ends. Long transitions that contain codeable intermediary motions/positions were coded as separate behaviors.*

***Lying***- This code captures all instances in which the infant is lying either on the front or on the back or side or rolling over. Although infants sitting in rockers, car seats, etc may not be fully upright, these instances were coded as sitting, rather than lying on the back, unless the infant was extended nearly horizontally.

*●Still-when the infant did not move, the lying was coded as still.*

*●Roll over*- when the infant rolled over- whether rolling from front to back, back to front, or rolling for an extended time on his/her side. The threshold for this code was 90 degrees.

*●Kick/Leg movement*- when the infant was lying and displayed any medium to large gross motor movement of the legs. This included clear leg limb extensions caused by imprecise muscle flexing or twitching provided that the movements were prominent or repetitive.

***Sitting***- The infant was coded as sitting whenever s/he was in an upright position and supported on the bottom/legs. Sitting on a lap, in a swing, car seat, or any other apparatus is included in this code. This code also includes instances in which the infant was sitting on knees in an upright position and not supported by the hands. (Instances in which the infant is strapped to the parent were coded as *Held* and not *Sit.*)

- *Independent-* instances in which the infant was seated anywhere without additional support. Instances in which the infant was sitting on his/her hip/bottom with weight supported by one or both arms and the hip/bottom were included in this modifier. This modifier does not include sitting on the mom’s lap or in a seat designed to assist the position (such as a baby swing or a walker, which were coded as *sit: assisted other*).
- *Assisted mom-* infant sits with support (typically on trunk) from mother.
- *Assisted other-* infant sits, typically in a seat with back support. Instances where an infant is in a walker and is stationary with the feet not supporting his/her weight fall under this modifier. Also, all instances in which the infant wass seated in a high chair or a swing with a back. (An infant sitting at the top of a slide, was coded *sit: independent*)
- *Rock/Lean/Bounce* – when the infant is sitting independently or sitting on another chair or on a swing, and the infant shows prominent movement of posture or position. Examples include rocking back and forth or bouncing up and down in a crawl position or if the infant is in an assisted seat or walker and initiates rocking, leaning, or bouncing movements. The key to this code is repetitive movements. However, instances in which the *mother* bounces the infant fall under the maternal stimulation code *rock/jiggle.* Furthermore, instances of limb movement while the trunk is stationary do not fall under this code and instead are covered under the category *Reaching and Extending*.

***Crawling***- This code captures all instances in which the infant locomotes by some means other than walking. This includes the typical hands and knees version of crawling, but is not limited to such expressions of crawling. Scooting or any other means by which the infant manages to pull him/herself around (aside from walking) qualifies for this code. It also includes stationary crawling position.

***Standing***- The infant was coded as standing when s/he stands upright on the feet, without walking. This may occur with or without the support of an object or adult.

- *Independent-* infant stands without holding onto his/her mother or any object (e.g., chair, couch, table edge). Instances in which the infant is merely resting a hand on an object (a table, for example), and not using it for assistance in the stand position fall under this code
- *Assisted mom-* infant stands and the mother assists by holding the infants hands or trunk
- *Assisted other-* infant stands while holding onto something for support, such as a table, chair, etc. This modifier also captures instances in which an infant is in a seat or walker and both feet are on the ground, and are supporting the infant’s weight (if no weight is supported, was coded as *sit: assisted other*). If the infant is clearly leaning on an object such as a table, and using its trunk, but not hands for support, this falls under the *assisted other* modifier. However, if the infant merely has a hand on a table, but is not using the table to assist in standing, this should be recorded as *stand: independent*.
- *Rock/Lean/Bounce* – when the infant is standing independently or assisted standing, the infant shows prominent movement of posture or position. Examples include rocking back and forth or bouncing up and down in a crawl position. The key to this code is repetitive movements. If the infant is in an assisted seat or walker and initiates rocking, leaning, or bouncing movements, use this code. However, instances in which the mother bounces the infant fall under the maternal stimulation code *rock/jiggle.* Furthermore, instances of limb movement while the trunk is stationary do not fall under this code and instead are covered under the category *Reaching and Extending*.

***Reaching***- This code captures all instances in which the infant extends the arm to grasp/get an object or to offer or to show an object. The beginning of a reach is coded when the first movement in the service of the reach is evident, even if this movement is postural or in the opposite direction of the reach. The end of the reach is coded when the infant makes contact with the object or gives up reaching for the object (note that successful grasping isn’t necessary to receive a reach code). A reach can be coded without large scale arm movement, if the arms are extended and clearly straining toward an object or a person. Reaching can include both hitting and retracting from the object to hit it again in the same place or one very close to it.

*● To grasp or to touch the object -* This code applies to the infant’s arm extension to grasp or to touch toy or an object

*● To offer or to show the objects* – This code applies to the infant’s arm extension to offer or show a toy or an object to the mother.

● *Other* – when the infant’s arm reaching is not clearly toward an object or a person, those kind of arm extension is coded as other.

***Pointing*** – Points for which both arm and finger are extended in the direction of the object of interest. Two- and three-month-old infants were more likely to point if they were alert and attentive, and their pre-pointing is more like a spontaneous expression of interest or attention than a direct communication about the object. After five months, pointing is used instrumentally to touch or tap objects while exploring, but it is not until about ten months that infants use pointing in communicative ways. After nine months, index finger pointing is incidental that is, an outstretched and index finger is used to refer to an object in visual space.

**Maternal Play Type (Frame) and Interactive Behavior**

*This category of codes was designed to capture all mother-infant dyadic play types and maternal play behaviors within the play type (frame). The coding process included two hierarchical steps. First, the coder identified specific play frames and then coded specific maternal behaviors within the identified frame. In identifying specific play type, coders recorded real-time onsets and offsets of play type and then conducted microanalytic coding of maternal behavioral sequences within the frame.*

**Mother-Infant Play Type (Frame)**

***Object Play with Infant (Guided object play)*** - This code refers to instances in which the mother plays with an object jointly with the infant. This is coded when mother take an active role in demonstrating and scaffolding the infant’s use of objects. Mother’s actions are directed toward demonstration of an object’s properties or supporting an object. Mother or infant may be holding an object. This can include tossing a toy back and forth or playing keys on a piano together. This code was used whenever a mother and infant both made play-related contact involving an object, except when such contact was blatantly accidental.

***Motor/social Play (Not-guided object play)*** - This code captures all instances in which the mother plays using gestures alone (i.e. no toys). This can include face-to face play, patty-cake, clapping, or playing peek-a-boo with one’s hands and physical-motor play. The topic of communication is the participants themselves with no intervening objects. Presenting hands in an act of encouragement should be coded as “motor play.”

***Passive Play (not-guided object play)*** - This is coded when the infant plays with objects without mother’s direct assistance but with her ongoing visual attention on the infant or providing postural support. However, mother does not touch the object or act on it in any direct manner. The infant is holding an object.

**Maternal Specific Behavior**

***Manipulation/Direction*** – This code refers to any sort of behavior to use, move, arrange, operate, play or control a toy or an object by the hands or in a skillful manner. This code includes mothers’ pretending behavior to show how to play by using a toy. For example, the mother hugs the teddy bear or greets or feeds the doll. Rolling a ball or turning pages of a book can be coded as manipulation. Mothers show or demonstrate how a toy works by holding a toy up or out towards the infant while looking at, gesturing, or verbalizing to the infant.

***Offering an object*** – offering the infant a toy by handing a toy or an object towards the infant (within arms reach); rearranging or setting toy down in front of the partner or on play partner’s lap; throwing or kicking a ball directly to the partner. It may or may not release the toy if the infant tries to accept the toy.

***Point***- This code refers to instances in which the mother points to direct the infant’s attention to something. A point can involve the use of more than one finger.

***Take/shift object***- This code should be applied whenever the mother removes a toy from the infant or adjusts or shifts a toy that the infant is playing with. Moving a toy so it is within the infant’s reach should not receive this code; instead it should be coded as “offer.”

***Hold object***- This code is designed to capture any other activity the mother does with an object including passively holding it.

***Responding*** – This code refers to any sort of behavior which imitates infant’s behavior or accepts or acknowledges the infant’s offered /shared toy or initiated any behavior.

***Affectionate touch***- This code refers to any sort of touch or behavior which primarily conveys affection, including tickles, nuzzles, kisses, stroking, and hugging. Additionally, stroking of the infant’s hair in the absence of a hair piece or brush should be coded as affectional.

***Gross motor stimulation***- This code captures all instances in which the mother moves the infants limbs so as to mimic infant gross motor behavior such as kicking or flailing the arms. Moving the limbs in the service of some activity which falls under another stimulation code (e.g. *Caretaking*, *Readjusting Position*) should not be coded as *Gross motor stimulation*. Restraining the infant’s arms/legs or preventing them from moving can, in some instances, be coded as “gross motor stimulation.”

***Assist locomotion/position/manipulation***- This code should be applied whenever the mother holds the infant in such a way to facilitate standing, walking, etc. A common instance of this code occurs when a mother supports hands of a young infant so s/he can stand or walk before s/he is able to do so independently. It occurs when a mother manipulates a toy with infant’s hand to teach or show how to use or manipulate a toy. This code can be interrupted by other codes in this category (e.g. affectional touch) that reflect a more pronounced physical exchange with the infant.

***Holding infant’s gross limb****- to assist infant’s locomotion or movement, the mother holds infant’s arms, torso, or legs.*

***Assisting infant’s fine motor/hand****-to assist infant’s manipulation or how to use the object, the mother hold the infant’s hand or fingers.*

***Shift infant***- This code refers to instances in which the mother repositions the infant. This might include picking the infant up from sitting to standing position, or shifting the infant closer to a toy. Little to no effort should be displayed by the infant, thus if the infant’s feet clearly leave the ground a shift should be coded. Minor adjustments with little effect on the infant’s position (e.g. when a mother is holding an infant and readjusts her grip on the infant or hikes the infant up slightly) should not be coded.

***Lift up***- This code should be applied to instances when the mother lifts the infant into the air as a kind of motor play type. Brief lifts that only serve the purpose of repositioning the infant should not be coded as *Lift up*, but instead should be coded as *Assist locomotion/position/manipulation*.

***Caretaking***- This code should be applied when the mother engages physically with the infant to perform a caretaking task, such as wiping nose, diapering, dressing or adjusting clothing, cleaning, feeding, burping, or protecting (i.e. removing something from mouth, etc.). A sustained caretaking act may involve minor interruptions without requiring that the code be turned on and off. The code should be turned off if the pauses last more than approximately one and a half seconds. For example, during feeding the code should be turned on only when the spoon is in contact with the mouth and therefore should turn off during refilling. Burping, and brief interludes of cleaning during feeding (e.g. wiping the mouth) should also be coded under caretaking feeding. Codes such as affectional that occur simultaneously with caretaking should trump the caretaking code.

***Rock/Jiggle***- This code refers to instances in which the mother rocks, jiggles, or in other waves moves the infant in some rhythmic way.

***Observing infant*** – This code refers to mother’s behavior to watch the infant’s face or infant’s activity.
