# Supplementary material for: Using Vector Autoregression Modeling to Reveal Bidirectional Relationships in Gender/Sex-Related Interactions in Mother–Infant Dyads
Source: Front Psychol. 2020 Aug 5;11:1507. doi: 10.3389/fpsyg.2020.01507 (PMC7419485; doi:10.3389/fpsyg.2020.01507)
Supplement: Supplementary file 2 [file Data_Sheet_2.docx]

Supplementary Material

Using vector-autoregressive modeling to reveal bidirectional relationships in sex-related interactions in mother infant dyads

**Elizabeth G. Eason, Nicole S. Carver, Damian G. Kelty-Stephen*, and Anne Fausto-Sterling**

*** Correspondence:** Damian G. Kelty-Stephen, foovian@gmail.com

**Supplementary Table 1.** Details of observational coding and reliability coding.

| *Infant Behaviors* | Agreement | Average kappa score |
| --- | --- | --- |
| Standing | 94% | 0.88 |
| Sitting | 88% | 0.809 |
| Lying | 89% | 0.771 |
| Babble | 93% | 0.866 |
| Cry | 89% | 0.674 |
| Reach | 77% | 0.638 |
| Crawling | 88% | 0.752 |
| *Play Frame (dyadic play)* |  |  |
| Passive | 72% | 0.485 |
| Motor/social | 81% | 0.764 |
| Object play | 88% | 0.762 |
| *Maternal Behaviors* |  |  |
| Rocking/Jiggling | 94% | 0.9 |
| Lifts Infant | 88% | 0.803 |
| Assists locomotion | 82% | 0.7 |
| Stimulates gross motor activity | 85% | 0.768 |
| Shifts Infant | 74% | 0.659 |
| Holds object | 69% | 0.5 |
| Points to object | 88% | 0.8 |
| Offers object | 81% | 0.654 |
| Manipulates object | 81% | 0.724 |
| Infant-Directed Speech | 71% | 0.606 |
| Affectionate Touch | 94% | 0.896 |
