# Supplementary material for: Using Vector Autoregression Modeling to Reveal Bidirectional Relationships in Gender/Sex-Related Interactions in Mother–Infant Dyads
Source: Front Psychol. 2020 Aug 5;11:1507. doi: 10.3389/fpsyg.2020.01507 (PMC7419485; doi:10.3389/fpsyg.2020.01507)
Supplement: Supplementary file 3 [file Data_Sheet_3.docx]

Supplementary Material

Using vector-autoregressive modeling to reveal bidirectional relationships in sex-related interactions in mother infant dyads

**Elizabeth G. Eason, Nicole S. Carver, Damian G. Kelty-Stephen*, and Anne Fausto-Sterling**

*** Correspondence:** Damian G. Kelty-Stephen, foovian@gmail.com

**Supplementary Data 2.** **Example R script for running VAR and IRF procedures**

library(vars) #begin by invoking the library “vars”

data<-read.csv(“inputfilename.csv”) #read in the data file.

varout<-VAR(data,p=1)#where data is a data.frame, and p = the number of lags

#see help(VAR) for more details of other input arguments

arch.test(varout) #autoregressive conditional heteroscedasticity test. This is a test for #heteroscedasticity for multivariate series, and it applies here to test whether the residuals of the #VAR model are heteroscedastic.

serial.test(varout) #test for serial correlations

#Both of the above tests should be nonsignificant, as we do not want to reject the null hypotheses of #homoscedasticity and independence across time

irfout<-irf(varout)#generates the IRFs for vars.

plot(irfout)# plots multiple panels of pairwise IRFs

#A word on ordering variables for conservative estimation of IRFs:

#Due to the Cholesky-decomposition method, order of variables in the VAR output influence the #orthogonalized form of the residuals. Note, this is not to say that order of variables matters to the #coefficients of the VAR. Order the variables as you like in your data frame, and you will get the #same coefficients for the VAR. The order only matters for the IRF because it relies on #orthogonalizing and because the Cholesky-decomposition method of orthogonalizing is an iterative #process that works from the first column to the next, etc. to produce elements of a diagonal matrix #in column-by-column sequence.

#What this means in practice is that, for any given running of the irf() code, you should only use the #irf for the last-entered (i.e., right most) variable in the dataframe you entered into the VAR() #function is going to get the most conservative estimates of is effect as an impulse variable. All non-#right-most variables are best examined only as response variables.

#So, when you plot IRFs, you may only want the last set of plots for each statement:

plot(irfout)

#Hence, after typing “plot(irfout),” there will be a prompt to press ENTER to see plots for each #successive variable’s effect as an impulse. Due to Cholesky decomposition, you may only want to #interpret the last of these plots, which involves pressing ENTER repeatedly until you have the last #plot.

#It is cumbersome, but if you want to have the most conservative estimates for a given variable's role #as an impulse variable, you should run the VAR with that given variable as the right-most, last-#entered variable. If you want to have the most conservative estimates for all variables' effects as #impulse then you need to run the VAR as many times as you have variables, each with one of the #variables entered last.

#For instance, if you have a data frame with three columns, then the full set of VARs and IRFs to be #run are as follows.

#To test the third column's effect as an impulse:

varout3<-VAR(data[,c(1,2,3)])

irfout3<-irf(varout3)

#To test the second column's effect as an impulse:

varout2<-VAR(data[,c(1,3,2)])

#or, equivalently

varout2<-VAR(data[,c(3,1,2)])

irfout2<-irf(varout2)

#To test the first column's effect as an impulse:

varout1<-VAR(data[,c(2,3,1)])

#or

varout1<-VAR(data[,c(3,2,1)])

irfout1<-irf(varout1)

Note that, although varout1, varout2, and varout3 will all be equivalent, irfout1, irfout2, and irfout3 will not be.
