# Supplementary material for: Using Vector Autoregression Modeling to Reveal Bidirectional Relationships in Gender/Sex-Related Interactions in Mother–Infant Dyads
Source: Front Psychol. 2020 Aug 5;11:1507. doi: 10.3389/fpsyg.2020.01507 (PMC7419485; doi:10.3389/fpsyg.2020.01507)
Supplement: Supplementary file 4 [file Data_Sheet_4.docx]

Supplementary Material

Using vector-autoregressive modeling to reveal bidirectional relationships in sex-related interactions in mother infant dyads

**Elizabeth G. Eason, Nicole S. Carver, Damian G. Kelty-Stephen*, and Anne Fausto-Sterling**

*** Correspondence:** Damian G. Kelty-Stephen, foovian@gmail.com

**Supplementary Data 2.** **List of significant effects resulting from VAR and IRF in Table 2 detailing effects of maternal behavior on later infant behavior**

**Column 1 of Table 2: Effects of increasing Rocking or Jiggling Infant.** Mothers increasing the number of seconds they rocked or jiggled their infants preceded later subsequent decreases in the number of seconds girls spent crying. Mothers increasing the number of occurrences that they were rocking or jiggling their infants preceded later subsequent increases in the number of occurrences that boys were in object play.

| Impulse-response forecasted relationship | Boys | Girls | Duration | Occurrence |
| --- | --- | --- | --- | --- |
| Mothers increasing rocking and jiggling infant preceded later decreases in infant crying | N | Y | Y | N |
| Mothers increasing rocking and jiggling infant preceded later increases in object play | Y | N | N | Y |

**Column 2 of Table 2: Effects of increasing Lifting Infants.** Mothers increasing the number of seconds they lifted their infants preceded later subsequent increases in the number of seconds boys spent sitting with object support. Mothers increasing the number of seconds they lifted their infants preceded later subsequent decreases in the number of seconds boys spent in object play.

| Impulse-response forecasted relationship | Boys | Girls | Duration | Occurrence |
| --- | --- | --- | --- | --- |
| Mothers increasing lifting infants preceded later increases in sitting with object support. | Y | N | Y | N |

**Column 3 of Table 2: Effects of increasing Assisting Locomotion.** Mothers increasing the number of occurrences that they assisted locomotion preceded later subsequent increases in the number of occurrences that boys stood independently.

| Impulse-response forecasted relationship | Boys | Girls | Duration | Occurrence |
| --- | --- | --- | --- | --- |
| Mothers increasing assistance of locomotion preceded later increases in standing independently. | Y | N | Y | N |

**Column 4 of Table 2: Effects of increasing Stimulation of Gross-Motor Locomotion.** Mothers increasing the number of seconds they spent stimulating gross-motor behavior preceded later subsequent decreases in the number of seconds boys spent standing with mother help. Mothers increasing the number of occurrences that they stimulated gross-motor behavior preceded later subsequent increases in the number of occurrences that boys lied still as well as the number of occurrence of boys engaging in all lying-down behaviors (Lie_All_). Mothers increasing the number of occurrences that they stimulated gross-motor behavior preceded later subsequent increases in the number of occurrences that boys engaged in passive play, motor-social play, and object play. Mothers increasing the number of seconds spent stimulating gross-motor behavior preceded later subsequent increases in the number of seconds that girls engaged in motor-social play.

| Impulse-response forecasted relationship | Boys | Girls | Duration | Occurrence |
| --- | --- | --- | --- | --- |
| Mothers increasing stimulation of gross-motor locomotion preceded later decreases in standing with mother help | Y | N | Y | N |
| Mothers increasing stimulation of gross-motor behavior preceded later increases in all lying. | Y | N | N | Y |
| Mothers increasing stimulation of gross-motor behavior preceded later increases in lying still | Y | N | N | Y |
| Mothers increasing stimulation of gross-motor behavior preceded later increases in passive play | Y | N | N | Y |
| Mothers increasing stimulation of gross-motor behavior preceded later increases in motor-social play | Y | N | N | Y |
| Mothers increasing stimulation of gross-motor behavior preceded later increases in motor-social play | N | Y | Y | N |
| Mothers increasing stimulation of gross-motor behavior preceded later increases in object play | Y | N | N | Y |

**Column 5 of Table 2: Effects of increasing Shifting of Infants.** Mothers increasing the number of seconds they spent shifting infant preceded later subsequent decreases in the number of seconds boys spent sitting with object support. Mothers increasing the number of occurrences that they shifted their infant preceded later subsequent increases in the number of occurrences that boys sit independently and that boys crawled.

| Impulse-response forecasted relationship | Boys | Girls | Duration | Occurrence |
| --- | --- | --- | --- | --- |
| Mothers increasing shifting infant preceded later decreases in sitting with object support | Y | N | Y | N |
| Mothers increasing shifting infant preceded later increases in sitting independently | Y | N | N | Y |
| Mothers increasing shifting infant preceded later increases in crawling | Y | N | N | Y |

**Column 6 of Table 2: Effects of increasing Holding Objects.** Mothers increasing the number of seconds they spent holding objects preceded later subsequent increases in the number of seconds girls spent standing independently. Mothers increasing the seconds spent [and the number of occurrences that they are] holding objects preceded later subsequent increases in the number of seconds that girls spend [and the number of occurrences that girls are] babbling. Mothers increasing the number of seconds they spent holding objects preceded later subsequent increases in the number of seconds boys spent in passive play.

| Impulse-response forecasted relationship | Boys | Girls | Duration | Occurrence |
| --- | --- | --- | --- | --- |
| Mothers increasing holding objects preceded later increases in standing independently | N | Y | Y | N |
| Mothers increasing holding objects preceded later increases in babbling | N | Y | Y | Y |
| Mothers increasing holding objects preceded later increases in passive play | Y | N | Y | N |

**Column 7 of Table 2: Effects of increasing Pointing to Object.** Mothers increasing the number of seconds they spent pointing to objects preceded later subsequent increases in the number of seconds girls spent standing with object support. Mothers increasing the number of seconds they spent [and the number of occurrences that they were] pointing to objects preceded later subsequent decreases in the number of seconds girls spent [and the number of occurrences that they were] standing with mother help. Mothers increasing the seconds spent pointing to objects preceded later subsequent increases in the number of seconds that boys spent standing independently, and mothers increasing the number of occurrences that they were pointing to objects preceded later subsequent increases in the number of occurrences of girls standing independently. Mothers increasing the number of occurrences that they were pointing to objects preceded later subsequent decreases in the number of occurrences of girls sitting with mother help. Mothers increasing the number of seconds spent pointing to objects preceded later subsequent decreases in the number of seconds boys spent sitting independently. Mothers increasing the number of seconds spent pointing to objects preceded later subsequent increases in the number of seconds boys spent sitting independently. Mothers increasing the number of seconds spent pointing to objects preceded later subsequent increases in the number of seconds girls spent reaching. Mothers increasing the number of occurrences that they were pointing to objects preceded later subsequent decreases in the number of occurrences of boys reaching. Mothers increasing the number of occurrences that they were pointing to objects preceded later subsequent increases in the number of occurrences of boys crawling. Mothers increasing the number of occurrences that they were pointing to objects preceded later subsequent decreases in the number of occurrences of girls engaging in motor-social play.

| Impulse-response forecasted relationship | Boys | Girls | Duration | Occurrence |
| --- | --- | --- | --- | --- |
| Mothers increasing pointing to objects preceded later increases in standing with object support | N | Y | Y | N |
| Mothers increasing pointing to objects preceded later increases in standing with mother support | N | Y | Y | Y |
| Mothers increasing pointing to objects preceded later increases in standing independently | Y | N | Y | N |
| Mothers increasing pointing to objects preceded later increases in standing independently | N | Y | N | Y |
| Mothers increasing pointing to objects preceded later decreases in sitting with mother help | N | Y | N | Y |
| Mothers increasing pointing to objects preceded later decreases in sitting independently | Y | N | Y | N |
| Mothers increasing pointing to objects preceded later increases in lying still | Y | N | N | Y |
| Mothers increasing pointing to objects preceded later increases in reaching | N | Y | Y | N |
| Mothers increasing pointing to objects preceded later decreases in reaching | Y | N | N | Y |
| Mothers increasing pointing to objects preceded later increases in crawling | Y | N | N | Y |
| Mothers increasing pointing to objects preceded later decreases in motor-social play | N | Y | N | Y |

**Column 8 of Table 2: Effects of increasing Offering Objects.** Mothers increasing the number of occurrences they spent offering objects preceded later subsequent increases in the number of occurrences that girls were standing with mother support. Mothers increasing the seconds spent offering objects preceded later subsequent increases in the number of seconds that boys spend sitting with object support. Mothers increasing the number of occurrences they spent offering objects preceded later subsequent decreases in the number of occurrences that boys were sitting independently. Mothers increasing the number of occurrences they spent offering objects preceded later subsequent decreases in the number of occurrences that girls were reaching.

| Impulse-response forecasted relationship | Boys | Girls | Duration | Occurrence |
| --- | --- | --- | --- | --- |
| Mothers increasing offering objects preceded later increases in standing with mother help | N | Y | N | Y |
| Mothers increasing offering objects preceded later increases in sitting with object support | Y | N | Y | N |
| Mothers increasing offering objects preceded later decreases in sitting independently | Y | N | N | Y |
| Mothers increasing offering objects preceded later decreases in reaching | N | Y | N | Y |

**Column 9 of Table 2: Effects of increasing Manipulating Object.** Mothers increasing the number of occurrences they were manipulating objects preceded later subsequent decreases in the number of occurrences that boys were sitting with mother help. Mothers increasing the seconds spent offering objects preceded later subsequent decreases in the number of seconds that boys spend in all types of lying and specifically lying still.

| Impulse-response forecasted relationship | Boys | Girls | Duration | Occurrence |
| --- | --- | --- | --- | --- |
| Mothers increasing manipulating objects preceded later decreases in sitting with mother help | Y | N | N | Y |
| Mothers increasing manipulating objects preceded later decreases in all lying behavior | Y | N | Y | N |
| Mothers increasing manipulating objects preceded later decreases in lying still | Y | N | Y | N |

**Column 10 of Table 2: Effects of increasing Speech to Infant.** Mothers increasing the number of seconds they spent speaking to infants preceded later subsequent decreases in the number of seconds that boys were sitting with object support. Mothers increasing the number of occurrences they were manipulating objects preceded later subsequent decreases in the number of occurrences that girls were engaged in types of lying and specifically lying still. Mothers increasing the number of seconds they spent speaking to infants preceded later subsequent decreases in the number of seconds that girls were reaching. Mothers increasing the number of occurrences they were manipulating objects preceded later subsequent increases in the number of occurrences that girls were engaged in passive play and object play.

| Impulse-response forecasted relationship | Boys | Girls | Duration | Occurrence |
| --- | --- | --- | --- | --- |
| Mothers increasing speech to infants preceded later decreases in sitting with object support | Y | N | Y | N |
| Mothers increasing speech to infants preceded later decreases in all lying behavior | N | Y | N | Y |
| Mothers increasing speech to infants preceded later decreases in lying still | N | Y | N | Y |
| Mothers increasing speech to infants preceded later decreases in reaching | N | Y | Y | N |
| Mothers increasing speech to infants preceded later increases in passive play | Y | N | N | Y |
| Mothers increasing speech to infants preceded later decreases in object play | Y | N | N | Y |

**Column 11 of Table 2: Effects of increasing Affectionate Touch.** Mothers increasing the number of seconds they spent providing affectionate touch preceded later subsequent increases in the number of seconds that boys were standing with mother help. Mothers increasing the number of seconds they spent providing affectionate touch preceded later subsequent decreases in the number of seconds that boys were sitting with mother help. Mothers increasing the number of seconds they spent providing affectionate touch preceded later subsequent increases in the number of seconds that boys were lying still. Mothers increasing the number of occurrences they were manipulating objects preceded later subsequent decreases in the number of occurrences that girls were reaching.

| Impulse-response forecasted relationship | Boys | Girls | Duration | Occurrence |
| --- | --- | --- | --- | --- |
| Mothers increasing affectionate touch preceded later increases in standing with mother help | Y | N | Y | N |
| Mothers increasing affectionate touch preceded later decreases in sitting with mother help | Y | N | Y | N |
| Mothers increasing affectionate touch preceded later increases in lying still | Y | N | Y | N |
| Mothers increasing speech to infants preceded later decreases in reaching | N | Y | N | Y |
