# Supplementary material for: Using Vector Autoregression Modeling to Reveal Bidirectional Relationships in Gender/Sex-Related Interactions in Mother–Infant Dyads
Source: Front Psychol. 2020 Aug 5;11:1507. doi: 10.3389/fpsyg.2020.01507 (PMC7419485; doi:10.3389/fpsyg.2020.01507)
Supplement: Supplementary file 5 [file Data_Sheet_5.docx]

Supplementary Material

Using vector-autoregressive modeling to reveal bidirectional relationships in sex-related interactions in mother infant dyads

**Elizabeth G. Eason, Nicole S. Carver, Damian G. Kelty-Stephen*, and Anne Fausto-Sterling**

*** Correspondence:** Damian G. Kelty-Stephen, foovian@gmail.com

**Supplementary Data 3.** **List of significant effects resulting from VAR and IRF in Table 3 detailing effects of infant behavior on later maternal behavior**

**Column 1 of Table 3: Effects of increasing Lie_All_.** Boys increasing the number of seconds spent in Lie_All_ behaviors preceded later subsequent increases in the number of seconds mothers spent stimulating gross-motor behavior. Also, boys increasing the number of occurrences of their Lie_All_ behaviors preceded later increases in the number of occurrences that mothers held objects for them.

| Impulse-response forecasted relationship | Boys | Girls | Duration | Occurrence |
| --- | --- | --- | --- | --- |
| Increasing Lie_All_ preceded later increases in mother stimulating gross-motor behavior | Y | N | Y | N |
| Increasing Lie_All_ preceded later increases in mother holding objects | Y | N | N | Y |

**Column 2 of Table 3: Effects of increasing Lying Still.** Boys increasing the number of seconds spent lying still preceded later subsequent decreases in the number of seconds mothers spent stimulating gross-motor behavior. Girls increasing the number of seconds spent lying still preceded later subsequent decreases in the number of seconds mothers spent shifting the infant. Also, boys increasing the number of occurrences of lying still preceded later a subsequent decrease in the number of occurrence that mothers held objects for them. Girls increasing the number of seconds spent lying still preceded later subsequent decreases in the number of seconds mothers spent offering an object.

| Impulse-response forecasted relationship | Boys | Girls | Duration | Occurrence |
| --- | --- | --- | --- | --- |
| Increasing Lie_Still_ preceded later decreases in mother stimulating gross-motor behavior | Y | N | Y | N |
| Increasing Lie_Still_ preceded later decreases in mother shifting infant | N | Y | Y | N |
| Increasing Lie_Still_ preceded later decreases in mother offering an object | Y | N | N | Y |
| Increasing Lie_Still_ preceded later decreases in mother holding objects | Y | N | N | Y |

**Column 3 of Table 3: Effects of increasing Reaching.** There were no significant effects of infants increasing reaching on subsequent maternal behaviors.

**Column 4 of Table 3: Effects of increasing Crawling.** Girls increasing the number of occurrences of their crawling preceded later subsequent decreases in the number of occurrences that mothers rocked or jiggled their infant. Girls increasing the number of occurrences of their crawling preceded later subsequent decreases in the number of occurrences that mothers assisted locomotion. Girls increasing the number of occurrences of their crawling preceded later subsequent decreases in the number of occurrences that mothers stimulated gross-motor behavior. Girls increasing the number of seconds spent crawling preceded later subsequent decreases in the number of seconds that mothers held objects for their infants. Boys increasing the number of occurrences of crawling preceded later subsequent increases in the number of occurrences that mothers held objects for their infants. Boys increasing the number of seconds spent crawling preceded later subsequent increases in the number of seconds that mothers pointed to objects. Girls increasing the number of seconds spent crawling preceded later subsequent decreases in the number of seconds mothers spent offering an object. Girls increasing the number of occurrences of crawling preceded later subsequent decreases in the number of occurrences that mothers touched their infants affectionately.

| Impulse-response forecasted relationship | Boys | Girls | Duration | Occurrence |
| --- | --- | --- | --- | --- |
| Increasing Crawl preceded later decreases in mother rocking/jiggling their infant | N | Y | N | Y |
| Increasing Crawl preceded later decreases in mother assistance to locomotion | N | Y | N | Y |
| Increasing Crawl preceded later decreases in mother stimulating gross-motor behavior | N | Y | N | Y |
| Increasing Crawl preceded later increases in mother holding objects for their infants | N | Y | Y | N |
| Increasing Crawl preceded later decreases in mother holding objects for their infants | Y | N | N | Y |
| Increasing Crawl preceded later increases in mother pointing to objects | Y | N | Y | N |
| Increasing Crawl preceded later decreases in mother offering objects to their infants | N | Y | Y | N |
| Increasing Crawl preceded later decreases in mother’s affectionate touch | N | Y | N | Y |

**Column 5 of Table 3: Effects of increasing Sitting with Mother help.** Boys increasing the number of occurrences of Sit_M_ behaviors preceded later subsequent decreases in the number of occurrences that mothers held objects for them.

| Impulse-response forecasted relationship | Boys | Girls | Duration | Occurrence |
| --- | --- | --- | --- | --- |
| Increasing Sit_M_ preceded later decreases in mother holding objects for their infants | Y | N | N | Y |

**Column 6 of Table 3: Effects of increasing Sitting with Object Support.** Girls increasing the number of seconds that they sit with mother’s help produces subsequent increases in the number of seconds that mothers rock or jiggle them. Girls increasing the number of seconds that they sit with mother’s help produces subsequent increases in the number of seconds that mothers assist locomotion. Girls increasing the number of occurrences that they sit with mother’s help produces subsequent decreases in the number of occurrences of mothers holding objects for them. Girls increasing the number of seconds that they sit with mother’s help produces subsequent decreases in the number of seconds that mothers offer objects. Girls increasing the number of seconds that they sit with mother’s help produces subsequent decreases in the number of seconds that mothers manipulate objects.

| Impulse-response forecasted relationship | Boys | Girls | Duration | Occurrence |
| --- | --- | --- | --- | --- |
| Increasing Sit_O_ preceded later increases in mother rocking/jiggling their infant | N | Y | Y | N |
| Increasing Sit_O_ preceded later increases in mother assisting locomotion | N | Y | Y | N |
| Increasing Sit_O_ preceded later decreases in mother holding objects for their infant | N | Y | N | Y |
| Increasing Sit_O_ preceded later decreases in mother offering objects to infants | N | Y | Y | N |
| Increasing Sit_O_ preceded later decreases in mother manipulating objects | N | Y | Y | N |

**Column 7 of Table 3: Effects of increasing Sitting Independently.** Boys increasing the number of occurrences that they sat independently preceded later subsequent decreases in the number of occurrences that mothers assisted locomotion. Boys increasing the number of occurrences that they sat independently preceded later subsequent increases in the number of occurrences that mothers held objects for their infants. Boys increasing the number of seconds spent sitting independently preceded later subsequent increases in the number of seconds mothers spent pointing to objects. Also, girls increasing the number of seconds spend sitting independently preceded later subsequent decreases in the number of seconds mothers spent offering an object.

| Impulse-response forecasted relationship | Boys | Girls | Duration | Occurrence |
| --- | --- | --- | --- | --- |
| Increasing Sit_I_ preceded later decreases in mother assisting locomotion | Y | N | N | Y |
| Increasing Sit_I_ preceded later increases in mother holding objects for their infant | Y | N | N | Y |
| Increasing Sit_I_ preceded later increases in mother pointing to object | Y | N | Y | N |
| Increasing Sit_I_ preceded later decreases in mother offering objects | N | Y | Y | N |

**Column 8 of Table 3: Effects of increasing Standing with Mother help.** Boys increasing the number of seconds spent standing with mother help preceded later subsequent decreases in the number of seconds that mothers rocked and jiggled them.

| Impulse-response forecasted relationship | Boys | Girls | Duration | Occurrence |
| --- | --- | --- | --- | --- |
| Increasing Sit_M_ preceded later decreases in mother rocking/jiggling their infants | Y | N | N | Y |

**Column 9 of Table 3: Effects of increasing Standing with Object Support.** Girls increasing the number of occurrences that they stood with object support preceded later subsequent decreases in the number of occurrences that mothers stimulated gross-motor behavior. Boys increasing the number of occurrences that they stood with object support preceded later subsequent increases in the number of occurrences that mothers held objects for their infants. Girls increasing the number of seconds spent standing with object support preceded later subsequent decreases in the number of seconds mothers spent offering objects. Also, boys increasing the number of seconds spent standing with object support preceded later subsequent decreases in the number of seconds mothers spent speaking to infants.

| Impulse-response forecasted relationship | Boys | Girls | Duration | Occurrence |
| --- | --- | --- | --- | --- |
| Increasing Stand_O_ preceded later decreases in mother stimulating gross-motor behavior | N | Y | N | Y |
| Increasing Stand_O_ preceded later increases in mother holding objects for their infant | Y | N | N | Y |
| Increasing Stand_O_ preceded later increases in mother offering object | N | Y | Y | N |
| Increasing Stand_O_ preceded later decreases in mother speaking to infant | Y | N | Y | N |

**Column 10 of Table 3: Effects of increasing Standing Independently.** Boys increasing the number of occurrences of their standing independently preceded later subsequent decreases in the number of occurrences that mothers rocked or jiggled their infant. Boys increasing the number of occurrences of their standing independently preceded later subsequent decreases in the number of occurrences that mothers lifted their infant. Girls increasing the number of seconds that they spent standing independently preceded later subsequent decreases in the number of seconds that mothers spent assisting locomotion. Boys increasing the number of occurrences of their standing independently preceded later subsequent increases in the number of occurrences that mothers stimulated gross-motor behavior. Girls increasing the number of seconds spent standing independently preceded later subsequent decreases in the number of seconds that mothers shifted their infants. Girls increasing the number of seconds spent [and of occurrences] standing independently preceded later subsequent increases in the number of seconds [and of occurrences] that mothers offers objects to their infants. Boys increasing the number of seconds spent standing independently preceded later subsequent decreases in the number of seconds [but subsequent increases in the number of occurrences] that mothers manipulated objects. Boys increasing the number of occurrences of standing independently preceded later subsequent decreases in the number of occurrences that mothers touched their infants affectionately.

| Impulse-response forecasted relationship | Boys | Girls | Duration | Occurrence |
| --- | --- | --- | --- | --- |
| Increasing Stand_I_ preceded later increases in mother rocking/jiggling their infant | Y | N | N | Y |
| Increasing Stand_I_ preceded later increases in mother assistance of locomotion | Y | Y | Y | N |
| Increasing Stand_I_ preceded later increases in mother stimulation of gross-motor behavior | Y | N | N | Y |
| Increasing Stand_I_ preceded later decreases in mother shifting their infant | N | Y | Y | N |
| Increasing Stand_I_ preceded later decreases in mother offering objects | N | Y | Y | Y |
| Increasing Stand_I_ preceded later decreases in mother manipulation of objects | Y | N | Y | N |
| Increasing Stand_I_ preceded later increases in mother manipulation of objects | Y | N | N | Y |
| Increasing Stand_I_ preceded later decreases in mother affectionate touch | Y | N | N | Y |

**Column 11 of Table 3: Effects of increasing Babbling.** Girls increasing the number of occurrences that they babbled preceded later subsequent increases in the number of occurrences that mothers lift them.

| Impulse-response forecasted relationship | Boys | Girls | Duration | Occurrence |
| --- | --- | --- | --- | --- |
| Increasing Babble preceded later increases in mother lifting their infants | N | Y | N | Y |

**Column 12 of Table 3: Effects of increasing Crying.** Girls increasing the number of seconds they spent crying preceded later subsequent increases in the number of seconds that mothers spent holding objects for them.

| Impulse-response forecasted relationship | Boys | Girls | Duration | Occurrence |
| --- | --- | --- | --- | --- |
| Increasing Crying preceded later increases in mother holding objects | N | Y | Y | N |

**Column 13 of Table 3: Effects of increasing Motor-Social Play.** Girls increasing the number of seconds that they spent in motor-social play preceded later subsequent increases in the number of seconds that mothers spend lifting them.

| Impulse-response forecasted relationship | Boys | Girls | Duration | Occurrence |
| --- | --- | --- | --- | --- |
| Increasing Play_M-S_ preceded later increases in mother lifting their infants | N | Y | Y | N |

**Column 14 of Table 3: Effects of increasing Object Play.** Boys increasing the number of occurrences of object play preceded later subsequent increases in the number of occurrences of mothers stimulating gross-motor behavior**.** Boys increasing the number of second spent in object play preceded later subsequent decrease in the number of seconds mothers spent holding them.

| Impulse-response forecasted relationship | Boys | Girls | Duration | Occurrence |
| --- | --- | --- | --- | --- |
| Increasing Play_O_ preceded later increases in mother stimulation of gross-motor behavior | Y | N | N | Y |
| Increasing Play_O_ preceded later decreases in mother holding infant | Y | N | Y | N |

**Column 15 of Table 3: Effects of increasing Passive Play.** Boys increasing the number of occurrences of object play preceded later subsequent decreases in the number of occurrences of mothers stimulating gross-motor behavior.

| Impulse-response forecasted relationship | Boys | Girls | Duration | Occurrence |
| --- | --- | --- | --- | --- |
| Increasing Play_O_ preceded later decreases in mother stimulation of gross-motor behavior | Y | N | N | Y |
